# Circulating tumor DNA methylation detection as biomarker and its application in tumor liquid biopsy: advances and challenges

**DOI:** 10.1002/mco2.766

**Published:** 2024-11-09

**Authors:** Lingyu Li, Yingli Sun

**Affiliations:** ^1^ Central Laboratory & Shenzhen Key Laboratory of Epigenetics and Precision Medicine for Cancers National Cancer Center/National Clinical Research Center for Cancer/Cancer Hospital & Shenzhen Hospital Chinese Academy of Medical Sciences and Peking Union Medical College Shenzhen China

**Keywords:** biomarker, circulating tumor DNA, liquid biopsy, methylation, tumor

## Abstract

Circulating tumor DNA (ctDNA) methylation, an innovative liquid biopsy biomarker, has emerged as a promising tool in early cancer diagnosis, monitoring, and prognosis prediction. As a noninvasive approach, liquid biopsy overcomes the limitations of traditional tissue biopsy. Among various biomarkers, ctDNA methylation has garnered significant attention due to its high specificity and early detection capability across diverse cancer types. Despite its immense potential, the clinical application of ctDNA methylation faces substantial challenges pertaining to sensitivity, specificity, and standardization. In this review, we begin by introducing the basic biology and common detection techniques of ctDNA methylation. We then explore recent advancements and the challenges faced in the clinical application of ctDNA methylation in liquid biopsies. This includes progress in early screening and diagnosis, identification of clinical molecular subtypes, monitoring of recurrence and minimal residual disease (MRD), prediction of treatment response and prognosis, assessment of tumor burden, and determination of tissue origin. Finally, we discuss the future perspectives and challenges of ctDNA methylation detection in clinical applications. This comprehensive overview underscores the vital role of ctDNA methylation in enhancing cancer diagnostic accuracy, personalizing treatments, and effectively monitoring disease progression, providing valuable insights for future research and clinical practice.

## Graphical abstract

The origin of ctDNA in the bloodstream is derived from CTC, exosomes secreted by tumor cells, apoptotic tumor cells and necrotic tumor cells (A). ctDNA methylation status plays an important role in the occurrence and development of cancer and is a biomarker for tumor liquid biopsy. Therefore, the development and optimization of a variety of ctDNA methylation detection techniques (B) can realize their clinical applications in tumor precision diagnosis and treatment, including early screening and diagnosis of cancer, identification of clinical molecular subtypes, recurrence and minimal residual disease (MRD) monitoring, predicting treatment response and prognosis, assessing tumor burden, and tracing tissue source (C).

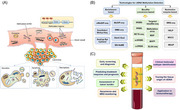

## 1 INTRODUCTION

**FIGURE 1:**
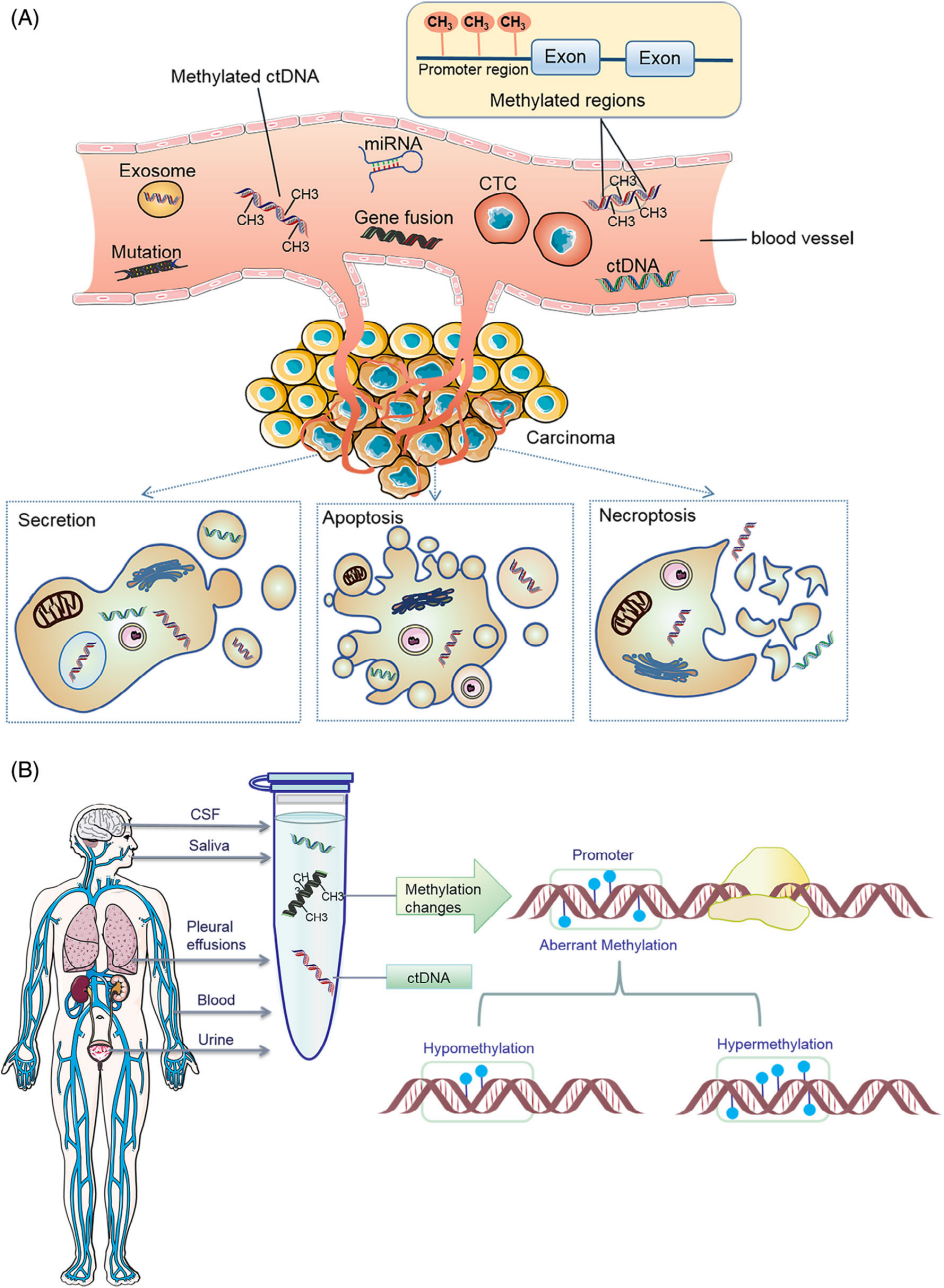
Sources and modes of circulating tumor DNA in body fluids. (A) Mode of circulating tumor cell cfDNA entry into the bloodstream. The origin of ctDNA in the bloodstream is derived from CTC, exosomes secreted by tumor cells, apoptotic tumor cells and necrotic tumor cells, which contain methylated ctDNA. (B) Body fluids as a source of circulating cell‐free tumor DNA. Schematic illustration of various bodily fluids that may contain ctDNA, including blood, urine, CSF, saliva, and pleural effusions. The presence of ctDNA in different body fluids is influenced by the location of the primary tumor and metastatic lesions. It is possible to identify methylation alterations associated with the cancer by analyzing ctDNA obtained from these circulating tumor materials in the body fluids. Aberrant methylation generally occurs in the promoter region including hypermethylation and hypomethylation states.

Cancer remains the primary cause of morbidity and mortality globally, emphasizing the importance of early detection and intervention for effective treatment and improved patient outcomes. Recent advancements in liquid biopsy have sparked significant interest due to its noninvasive nature and broad application potential.^1^ This innovative approach analyzes circulating analytes, including circulating nucleic acids (circulating tumor DNA [ctDNA] and cell‐free RNA [cfRNA]), circulating tumor cells (CTCs), tumor‐specific cell‐free DNA (cfDNA) methylation, and extracellular vesicles (EVs, mainly exosomes),^2^, ^3^ to assess cancer. Liquid biopsy technology can not only detect ctDNA, but also simultaneously analyze CTCs, exosomes, and microRNAs (miRNAs) and other biomarkers. The combined detection of multiple biomarkers provides a more comprehensive perspective for cancer diagnosis.^4^, ^5^ Among these, ctDNA mutation detection is primarily utilized for companion diagnosis and guiding therapy in advanced cancer stages, whereas CTC analysis, constrained by cell capture technology, is more suited for prognostic evaluation. The integration of tumor ctDNA and ctDNA methylation detection currently represents the most effective method for early cancer screening. ctDNA, a mixture of genetic material released into bodily fluids through apoptosis, necrosis, or active secretion, can be detected in the bloodstream,^6^ as shown in Figure 1A. Typically, the length of cfDNA double‐stranded fragments ranges from 150 to 200 base pairs, with concentrations in healthy adult plasma usually below 10 ng/mL.^7^ A specific subset of cfDNA, known as ctDNA, originates from cancer cells and circulates in the bloodstream.^8^ The short half‐life of cfDNA, combined with the noninvasive repeatability of liquid biopsies, renders ctDNA‐based analysis an effective tool for real‐time monitoring of tumor dynamics.^9^ Recent research has demonstrated the potential of ctDNA detection in early cancer diagnosis, treatment efficacy evaluation, and prognosis prediction.^10^, ^11^, ^12^ For instance, Shitara et al.^13^ identified that baseline ctDNA gene alterations could act as biomarkers for survival in metastatic colorectal cancer (CRC) patients treated with panitumumab and chemotherapy, suggesting their potential to guide optimal treatment decisions. Additionally, Li et al.^14^ developed MESA, a multimodal epigenetic sequencing analysis for noninvasive CRC detection, leveraging cfDNA's epigenetic properties. Bert Vogelstein's team revealed that the increased cfDNA in cancer patients’ blood primarily originates from white blood cells, not tumors or damaged normal tissues,^15^ suggesting a systemic effect of cancer on cell turnover or DNA clearance and providing new insights into cfDNA's origin and implications for liquid biopsy research. Currently, blood‐based tests are widely used, and ctDNA detection extends to various bodily fluids, such as pleural effusion, ascites, saliva, urine, feces, and cerebrospinal fluid (CSF),^16^, ^17^, ^18^ as summarized in Figure 1B, highlighting the diverse sources of ctDNA. In recent years, with the rapid development of high‐throughput sequencing technology and molecular biology research, the potential application of ctDNA in cancer diagnosis, treatment monitoring, and prognosis assessment has become increasingly prominent. Specifically, the methylation of ctDNA, as a significant form of epigenetic modification, has emerged as a focal point in cancer research.

Epigenetic mechanisms are pivotal in organismal development, with alterations potentially leading to cell transformation and malignancy. A versatile epigenetic testing method applicable to 1 mL plasma samples has been developed and used in a study involving 433 patients across 15 cancer types, enabling concurrent detection of histone modifications and DNA methylation, and facilitating assessment of key gene activities. This method lays the groundwork for personalized therapy.^19^ DNA methylation, a key epigenetic mechanism, regulates gene expression and maintains genome stability. Dysregulation of methylation is common across various tumor types, contributing to tumor development, progression, and metastasis.^20^, ^21^, ^22^, ^23^ The detection of ctDNA methylation, encompassing both the number and expression of methylation sites and the identification of 5‐hydroxymethylcytosine (5hmC).^24^ Methylation profiling of ctDNA has emerged as a potent tool for early detection, monitoring recurrence, and prognostic prediction in various cancers.^25^, ^26^, ^27^ The discovery that ctDNA methylation patterns mirror those in cancerous tissue suggests that circulating tumor‐derived DNA could serve as a promising source of biomarkers for liquid biopsy.^28^, ^29^ cfDNA methylation now identifies small cell lung cancer (SCLC) subtypes, marking a novel application in liquid biopsy.^30^, ^31^ Studies have revealed that SCLC‐I subtypes respond better to adjuvant immunotherapy combined with chemotherapy.^32^, ^33^ The discovery that hypermethylated STAT5A leads to regulatory suppression and immune cell depletion in squamous cell carcinomas underscores the significance of STAT5A's epigenetic regulation in immunosuppression.^34^ The recent breakthrough in identifying methylation markers within peripheral blood mononuclear cells (PBMCs) offer a novel direction for developing biomarkers.^35^, ^36^ Further, a comprehensive analysis of plasma cfDNA and genomic DNA from peripheral blood leukocytes in SCLC patients revealed that diminishing methylation in peripheral blood leukocytes enhances tumor specificity.^37^ ctDNA methylation is also effective in minimal residual disease (MRD) assessment in solid tumors.^38^, ^39^ The United States Food and Drug Administration (US FDA)’s approval of plasma methylated ctDNA as a screening tool for CRC underscores its efficacy.^40^ Methylation pattern changes in cfDNA are instrumental in diagnosing, staging, predicting prognosis, and detecting recurrence in breast cancer (BC)^41^ and ovarian cancer (OC).^8^ The discovery of 5hmC in ctDNA offers diagnostic and prognostic insights across various cancers.^42^, ^43^ Epigenetic markers emerge as superior early diagnostic biomarkers compared with genetic mutation screening.^44^, ^45^ ctDNA methylation stands out as a highly valuable method for cancer diagnosis and risk assessment, offering greater sensitivity and localization accuracy than gene mutation detection.^46^, ^47^ A prospective cohort study introduced ColonSecure,^48^ a blood‐based, noninvasive CRC detection method based on ctDNA‐specific methylation patterns, outperforming traditional markers. Additionally, a novel liquid biopsy strategy combining circulating free mitochondrial DNA (mtDNA) with ctDNA has shown to enhance cancer detection.^49^

Although numerous studies have highlighted the potential of ctDNA as a biomarker for the early detection and diagnosis of cancer, significant challenges remain. In early‐stage cancers, ctDNA levels are often low, and the genomic profile of the primary tumor may not be well‐defined, necessitating highly sensitive detection methods. Moreover, the variability of ctDNA biomarkers across individuals complicates the development of universally sensitive and applicable techniques.^1^ Whole‐genome bisulfite sequencing (WGBS) encompasses the broadest genomic coverage among DNA methylation detection methods, showing considerable promise for ctDNA methylation analysis. Our recent study introduced an improved ctDNA–WGBS technique capable of isolating minute quantities of ctDNA from plasma and precisely mapping genome‐wide methylation patterns.^50^ In view of the importance and challenges of ctDNA methylation detection in tumor liquid biopsy, this paper hopes to provide a valuable reference for researchers in related fields by reviewing the latest research progress and challenges of ctDNA methylation detection, and promote its widespread application in tumor liquid biopsy. This review synthesizes key findings from the latest research, which leverages sophisticated statistical analyses to assess ctDNA methylation's viability as an epigenetic biomarker for cancer diagnosis, prognosis, and therapeutic prediction, aiming to integrate these insights into clinical practice. Through comprehensive data examination, these investigations confirm ctDNA methylation's role as a highly sensitive and specific tumor marker, suitable for early cancer detection, ongoing treatment evaluation, and prognosis determination. Notably, while ctDNA methylation patterns may vary among patients with different cancers, certain DNA methylation alterations are consistent across various cancer types. This underscores the necessity of selecting specific detection markers tailored to each cancer type in clinical practice. The recent progress in ctDNA methylation research marks a significant stride forward, offering new avenues to address the medical community's current challenges. These advancements herald a shift toward more personalized, intelligent, and efficient precision medicine, enhancing our capacity to manage and treat cancer effectively. This review highlights the potential of ctDNA methylation as a highly sensitive and specific marker for cancer diagnosis and monitoring. By summarizing key findings from the latest research, we aim to synthesize the current understanding of ctDNA methylation's role in cancer and provide insights into its future clinical applications.

This review summarizes the challenges and advances in ctDNA methylation detection as a biomarker and its application in tumor liquid biopsy. First, we summarize the development and progress of ctDNA methylation detection, emphasizing its advantages as a liquid biomarker, and reviewing the main technical methods of ctDNA methylation detection. Second, we discuss the challenges faced in the application of ctDNA methylation. Subsequently, we delve into the recent advancements and clinical applications of ctDNA methylation in early cancer screening, identification of clinical molecular subtypes, monitoring of recurrence and MRD, prediction of treatment response and prognosis, assessment of tumor burden, and determination of tissue origin. Additionally, we explore the limitations of current technologies and future perspectives in this rapidly evolving field.

## 2 DEVELOPMENT AND PROGRESSION OF CTDNA METHYLATION DETECTION

ctDNA methylation is the most potential way among all liquid biopsy methods. The benefits of using ctDNA methylation as a liquid biomarker are primarily manifested in the following aspects: sensitivity and specificity. Methylation analysis has shown high sensitivity and specificity in detecting tumor recurrence or monitoring disease progression. For example, in some studies, the sensitivity of MRD detection based on methylation was significantly higher than that based on ctDNA mutations.^18^ This means that methylation analysis can more accurately identify patients with tumors, thereby helping with early diagnosis and treatment. *Early detection*: Methylation changes are early events in tumor development, so by detecting changes in ctDNA methylation, it may be possible to diagnose earlier in the course of tumor development. This is of great importance for improving treatment outcomes and patient survival rates. *Stability*: Compared with gene mutations, methylation markers are more stable. Gene mutations may be difficult to detect due to tumor heterogeneity or clonal evolution, while methylation patterns usually remain consistent throughout the course of tumor development. This makes methylation analysis a more reliable biomarker. *Ease of analysis*: Compared with mutation detection that requires searching for changes throughout the entire genome, methylation analysis may be easier to implement. Because it primarily focuses on changes in the promoter regions, which have known positions in the genome, it simplifies the analysis process. *Wide applicability*: Methylation as an epigenetic modification occurs in many types of cancer. Therefore, ctDNA‐based methylation detection may have a wider range of applicability, able to be used for the diagnosis and monitoring of various types of cancer. In summary, ctDNA methylation as a liquid biomarker for cancer has significant advantages in terms of sensitivity, specificity, early detection ability, stability, and ease of analysis. These advantages make methylation analysis have important application value in cancer diagnosis and treatment. However, it is worth noting that each biomarker has its limitations, and therefore, multiple factors should be considered in practical applications to choose the most suitable detection method.

**FIGURE 2:**
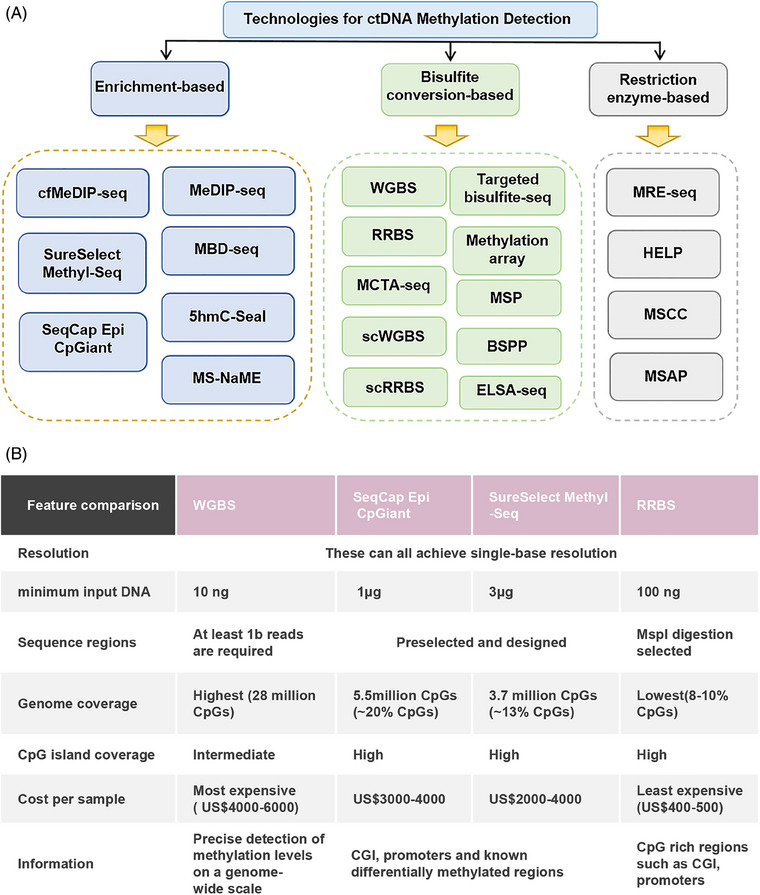
Technologies and platforms for ctDNA methylation detection and sequencing. (A) Technologies for ctDNA methylation detection. cfMeDIP‐seq, cell‐free methylated DNA immunoprecipitation sequencing; MBD, methyl‐CpG‐binding domain; Methyl‐Seq, methylation sequencing; SeqCap, sequence capture; MS‐NaME, methylation specific nuclease‐assisted minor‐allele enrichment; MSP, methylation‐specific PCR; scWGBS, single‐cell WGBS; scRRBS, single‐cell RRBS; BSPP, bisulfite padlock probes; ELSA‐seq, enhanced linear fragment amplification sequencing; MRE‐seq, methylation restriction enzyme digestion followed by sequencing; HELP, HpaII‐tiny fragment enrichment by ligation‐mediated PCR; MSCC, methyl‐sensitive cut counting; MSAP, methylation‐sensitive amplified polymorphism. (B) Common base resolution methylation sequencing platforms. The required quantity varies according to the protocols.

The importance of ctDNA in cancer diagnosis and treatment is increasingly recognized, and effective ctDNA isolation is a prerequisite for its analysis. Currently, there are several methods for ctDNA isolation, each with its unique advantages and limitations. Immunoprecipitation‐based methods have high specificity, but may not capture all types of ctDNA and are expensive; the collection method using blood collection tubes (BCT) is suitable for retrospective studies, but note that some BCT tubes may contain chemicals that affect DNA methylation patterns; magnetic bead‐based isolation methods are efficient and fast, suitable for high‐throughput processing, but the specificity of the magnetic beads may affect the recovery rate and purity of ctDNA; the utilization of commercial kits offers a convenient and user‐friendly approach, but their performance may vary depending on the sample type and quality. When choosing a ctDNA isolation method, consider the sample size, ctDNA purity and recovery rate, cost, and experimental objectives among other factors. Additionally, the ctDNA isolated needs to be converted to bisulfite for analysis of epigenetic markers, and care should be taken to choose the right kit and method for this step. In summary, there is no “best” ctDNA isolation method that is suitable for all situations, and researchers should choose the most appropriate method based on specific needs and experimental conditions. With the continuous development of technology, there is hope for the emergence of more efficient and specific methods for isolating and analyzing ctDNA in the future, further driving the development of cancer diagnosis and treatment. Analyzing ctDNA biomarkers, given their low abundance, necessitates highly sensitive and specific detection techniques. The technologies available for DNA methylation detection each have distinct advantages and limitations.^51^ These methods fall into two main categories: those based on bisulfite conversion and those that do not require bisulfite conversion (Figure 2A). In addition, there are methods based on polymerase chain reaction (PCR) and next‐generation sequencing (NGS).

### 2.1 Bisulfite conversion‐based methods

Bisulfite conversion‐based methods exploit the unique property that cytosine residues can be converted to uracil in genomic DNA, but methylcytosine remains unchanged after bisulfite treatment, facilitating the identification of 5‐methylcytosine (5mC) and cytosine.^52^ Techniques such as WGBS, SureSelect Methyl‐Seq, and SeqCap Epi CpGiant reduced representative bisulfite sequencing (RRBS)^53^ (Figure 2B), methylated CpG tandem amplification and sequencing (MCTA‐seq), and methylation arrays have been developed.^54^ These methods enable precise identification of DNA methylation changes at the single‐base level, though genomic coverage varies. Based on this, Shen et al.^55^ used a loop‐mediated isothermal amplification method based on thiol‐primed primers to achieve high sensitivity detection of ctDNA E‐Box methylation in tumor tissues and biological samples of renal cell carcinoma patients. Therefore, researchers are advised to select the most suitable technique based on the specific requirements of their study.

**FIGURE 3:**
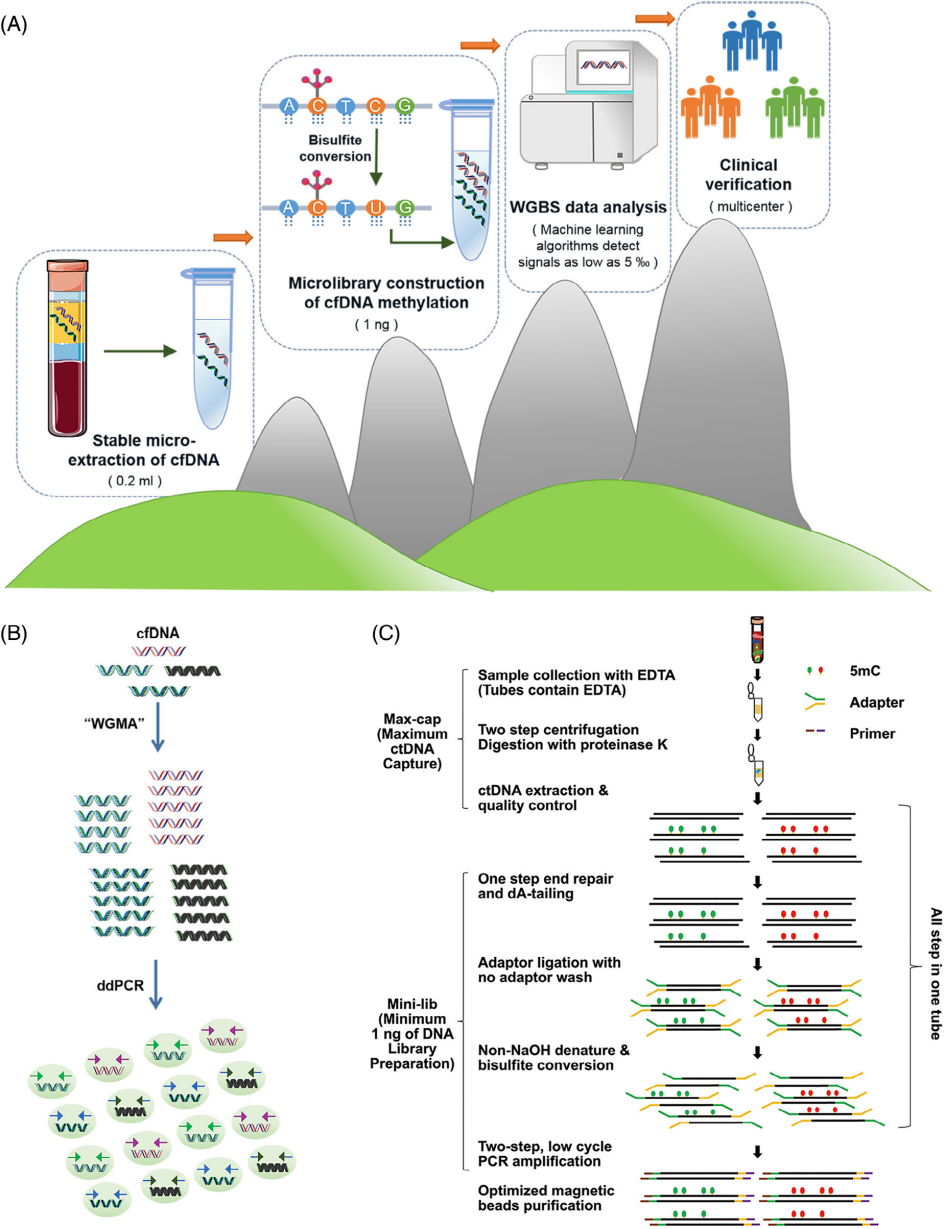
Innovative approaches for ctDNA analysis: improved WGBS method, genome‐wide methylation amplification with digital PCR, and whole‐genome 5mC methylation sequencing. (A) An improved ctDNA–WGBS method. First, cfDNA was stably microextracted from plasma, and then as low as 1 ng of input DNA was utilized for methylation microlibrary construction. Then, machine learning algorithm was used to analyze the WGBS data, which could detect as low as five out of 1000 of the signal, finally, clinical multicenter validation was performed. (B) cfDNA was amplified by genome‐wide methylation followed by digital PCR. WGMA, Whole‐genome methylation amplification; ddPCR, digital droplet polymerase chain reaction. (C) Whole‐genome methylation sequencing of 5mC in ctDNA. ctDNA is extracted from plasma. After purification, the ctDNA is ligated with an adapter and subjected to bisulfite conversion. PCR amplification is then performed on the fragments, which are subsequently captured using beads.^50^

Bisulfite sequencing emerges as the most dependable technique for DNA methylation analysis, offering precise quantification at the single‐base level.^56^ Addressing the challenges posed by bisulfite treatment, such as DNA damage and reduced sequence diversity, Liang et al.^57^ introduced ELSA‐seq. WGBS technology facilitates comprehensive identification of methylation sites across the genome.^6^ Our recent advancement in ctDNA–WGBS methodology enables the accurate determination of genome‐wide methylation patterns from a minimal volume of ctDNA, extracted from merely 200 µL of plasma, as shown in Figure 3A. This refined approach, utilizing as little as 1 ng of DNA for library preparation, achieves unbiased genome‐wide coverage and employs a comprehensive computational strategy to minimize background noise from low‐repeat and nontumor‐derived ctDNA. This innovation allows for early BC screening across multicenter patient cohorts with high specificity and sensitivity, developing highly precise biomarkers for distinguishing various cancer types and subtypes.^50^ Such advancements hold significant potential for clinical application. GRAIL has harnessed bisulfite sequencing combined with machine learning to develop a methylation pattern classification method, achieving 67% sensitivity and 99% specificity in detecting early‐stage disease across multiple cancer types.^58^ Huang et al.^59^ utilized WGBS to profile DNA methylation in small volumes of CSF ctDNA from children with medulloblastoma (MB), demonstrating its utility in pediatric oncology. However, the limitations of bisulfite conversion, particularly its inefficiency in capturing DNA prone to fragmentation during apoptosis,^60^ highlight the importance of methodological consideration in cfDNA methylation studies. Expanding upon this, Maggi and colleagues^61^ have developed a method for comprehensive cfDNA methylation analysis, producing high‐quality libraries with a 98% conversion rate, suitable for plasma‐based studies.

### 2.2 Nonbisulfite conversion methods

Nonbisulfite conversion methods for DNA methylation analysis include antibody enrichment and restriction endonuclease techniques. The antibody enrichment approach, particularly MeDIP (methylated DNA immunoprecipitation), uses a 5mC antibody to selectively enrich methylated DNA fragments, identifying methylation levels through fluorescence differences.^62^ This category encompasses sequencing methods like MeDIP‐Seq and MBD‐Seq,^63^ where the development of recombinant protein complexes with enhanced affinity is crucial. cfMeDIP‐seq, for instance, has shown high sensitivity in ctDNA methylation detection, effectively identifying and classifying various cancer types.^64^ Nuzzo et al.^65^ utilized cfMeDIP‐seq to detect ctDNA with 97% sensitivity and 100% specificity in a study on 69 individuals with renal carcinoma at different stages. Methyl‐sensitive restriction endonucleases, another approach, exhibit varying sensitivities to methylcytosine, aiding in methylation detection.^52^ However, their application is limited by the specificity of enzyme recognition sites, and methylation‐specific restriction endonucleases (MSRE) are costly and less effective in regions of intermediate methylation. Recently, Gouda et al.^66^ introduced a sequencing method targeting 32 specific CpG sites for methylation detection in CRC. Before clinical application, these methods necessitate extensive validation to ensure their reliability and effectiveness in diagnostics, underscoring the need for comprehensive testing across a broad range of conditions.

### 2.3 PCR and NGS methods

PCR and NGS‐based methodologies have been instrumental in assessing DNA methylation status, each with distinct advantages for ctDNA biomarker analysis. PCR‐based techniques, such as MSP,^67^ methylation‐sensitive high‐resolution melting analysis,^68^, ^69^ MethyLight PCR,^70^ one‐step methylation‐specific PCR (OS‐MSP),^71^ methylation‐specific PCR‐coupled liquid bead array,^72^ real‐time methylation‐specific PCR (RT‐MSP), and multiplex nested methylation‐specific PCR,^73^ are pivotal for quantifying ctDNA methylation. These methods, which utilize specific primers for methylated and nonmethylated DNA, are designed for separate PCR reactions targeting known sequences,^74^ thereby enhancing sensitivity and specificity. ddPCR emerges as a cutting‐edge technique, leveraging nanoscale water‐in‐oil droplet technology for the quantitative detection of DNA methylation (Figure 3B). This approach, notable for its high sensitivity, accuracy, and the ability to provide absolute quantification without a standard curve, is particularly suited for cancer liquid biopsy applications. ddPCR's simplicity, rapid setup, and minimal sample requirements, coupled with its remarkable sensitivity, offer significant advantages over NGS methods.^75^ Furthermore, ddPCR's lack of need for complex bioinformatics analysis, alongside its superior sensitivity compared with real‐time PCR (RT‐PCR), makes it an invaluable tool for detecting low‐abundance targets like methylated ctDNA with greater precision and accuracy.^76^ Building on this foundation, researchers have developed “TriMeth,” a tumor‐agnostic digital PCR assay targeting three CRC‐specific methylation markers (C9orf50, CLIP4, KCNQ5), demonstrating high specificity and sensitivity in a cohort of patients undergoing surgery for colorectal liver metastases (CRLM).^77^ These advancements underscore the evolving landscape of molecular diagnostics, where PCR and NGS‐based methods, particularly ddPCR, play a crucial role in enhancing the detection and analysis of ctDNA methylation, paving the way for more precise, efficient, and personalized cancer diagnostics and monitoring.

NGS methods, including MethylCap‐Seq,^78^ MeDIP‐Seq,^79^ and MBD‐seq,^80^ are pivotal for detecting methylation levels across multiple genes in ctDNA. In particular, Xin et al.^81^ used NGS technology to analyze the methylation patterns of tumor suppressor genes in ctDNA from pancreatic cancer patients and found that it has a broad prospect as a noninvasive detection and monitoring method. While pyrosequencing offers precise examination of methylated regions, its high cost limits widespread application. Methylation‐sensitive high resolution melting presents a cost‐effective, rapid, and accurate PCR‐based alternative. The MethyLight PCR technique, leveraging fluorescence‐labeled probes with bisulfite‐treated DNA, enables specific detection of DNA methylation patterns.^20^ Despite the potential of NGS for predicting cancer recurrence through ctDNA analysis, the complexity and expense of these methods pose challenges for routine clinical application. Addressing this, Jin et al.^82^ introduced a cost‐effective, single‐tube methylation‐specific quantitative PCR (mqMSP) method utilizing a panel of 10 methylation markers, capable of detecting as low as 0.05% tumor DNA in plasma. This approach enhances liquid biopsy's predictive accuracy for cancer recurrence, surpassing traditional methods and even the methylated Septin 9 (mSEPT9) assay in detecting early and precancerous polyps.^82^ The US FDA has approved Epi proColon and its updated version, Epi proColon 2.0, for CRC screening, utilizing DNA hypermethylation analysis.^83^ These tests, based on RT‐PCR for mSEPT9 DNA detection, offer higher diagnostic sensitivity than conventional markers and fecal occult blood testing.^83^, ^84^, ^85^ Innovative techniques like “CancerDetector,”^86^ which simulates collective methylation status across CpG sites, and GRAIL's prototype methylation technology, demonstrate the evolving landscape of cancer detection. These methods enable early‐stage identification and precise tumor origin determination,^87^ showcasing the potential of methylation analysis in enhancing cancer diagnostics. Furthermore, targeted and shallow genome sequencing, as developed by Nguyen et al.,^88^ integrates multiple analytical dimensions, offering a comprehensive and cost‐effective approach for early cancer detection and localization. This multimodal method, suitable for broad screening, underscores the shift toward more accessible and efficient diagnostic technologies. In summary, advancements in PCR and NGS‐based methylation analysis are revolutionizing cancer detection, moving toward more sensitive, specific, and cost‐effective methodologies. These developments hold promise for improving early diagnosis, guiding treatment decisions, and enhancing the precision of cancer treatment.

Current efforts to enhance the sensitivity of liquid biopsies for cancer are primarily focused on refining sequencing and in vitro analytical steps. However, the underlying challenge is the low abundance of ctDNA in blood samples. Martin‐Alonso et al.^89^ tackled this issue by developing a dual‐component priming agent that combines lipid nanoparticles and monoclonal antibodies with cfDNA, effectively reducing macrophage phagocytosis and nuclease degradation of cfDNA. This innovation led to a tenfold increase in ctDNA yield and improved the sensitivity of tumor analysis using ctDNA from less than 10–75% by administering the agent intravenously 1−2 h before sample collection. The agent's effect on cfDNA is transient, similar to that of contrast agents, necessitating further research to confirm its efficacy and tolerability in humans. Recent advancements in ctDNA technology have concentrated on enhancing sequencing sensitivity, identifying variations in methylated sites among individuals, and discovering cancer‐specific cfDNA methylation biomarkers. Analytical methods such as receiver operating characteristic curve analysis, *t*‐tests, and the Kaplan–Meier method have been employed for disease‐free survival (DFS) analysis, alongside Cox proportional hazards regression and CpG differential methylation analysis.^90^, ^91^, ^92^ These efforts aim to improve the accuracy and potential of ctDNA methylation detection, as summarized in Table 1. The utilization of marker combinations offers greater accuracy than individual markers. However, many current methylation detection methods are either too complex for routine clinical application or offer lower sensitivity than alternative ctDNA detection methods. This underscores the need for a streamlined process that ensures high sensitivity and specificity while maintaining broad applicability. Liquid biopsy technology has been made possible by the advancement of microfluidics and nanotechnology, enabling highly sensitive detection of circulating biomarkers such as ctDNA and miRNA.^93^, ^94^, ^95^ ctDNA methylation analysis and liquid biopsy technology have shown great potential for cancer diagnosis, prognosis monitoring, and treatment guidance. With the advancement of nanotechnology, microfabrication technology, and biosensing technology, these technologies will achieve higher sensitivity, specificity, and portability in the future, providing more effective tools for cancer management.^4^, ^5^, ^96^, ^97^

**TABLE 1:** Summary of analytical approaches for ctDNA methylation detection.

Cancer type	Application	Sample type	Sample size	Methodology	ctDNA methylation biomarkers	Main findings	References
CRC	Monitoring of recurrence	Plasma	Different stages of 130 patients	High‐throughput targeted DNA sequencing and random forest	SFMBT2, SGCG, ZNF568, ZNF671 and ZNF132	Sensitivity (87.5%) and specificity (94.12%)	^25^
BC	Early diagnosis	Plasma	BC: *n* = 123; healthy controls: *n* = 40	WGBS, ddPCR	15 ctDNA methylation markers	[AUC] = 0.967	^50^
LC	Early detection	Plasma	308 patients and 261 control	ELSA‐seq and MBS	–	Specificity of 96%	^57^
>50 cancer types	Early detection	Plasma	2482 patients (> 50 cancer types), 4207 controls	Bisulfite sequencing and tissue of origin localization classifier	–	Sensitivity of 67% and specificity of 99%	^58^
CRC	Monitoring of recurrence	Plasma	182 samples from 82 patients	mqMSP	10 different methylation markers	ctDNA was detected in 70% of patients, 8.0 months earlier than radiographic imaging	^82^
OPC	Monitoring of recurrence	Plasma	252 HNSCC patients	Q‐MSP	CALML5, DNAJC5G and LY6D	The methylation rates of CALML5, DNAJC5G and LY6D were 100%, 87.5% and 87.5%, respectively	^90^
CRC	Diagnose	Plasma	272 patients and 402 control	ddPCR	MYO1‐G	[AUC] = 0.94, sensitivity of 84.3% and specificity of 94.5%	^91^
HCC	Early screening	Serum	27 controls and 31 patients	Illumina 450 BeadChip and bisulfite sequencing.	DBX2 and THY1	Sensitivity of 88.89 % and specificity of 87.10%	^98^
LC	Early detection	Plasma	46 patients and 385 control	Genome‐wide cfDNA fragmentomes analysis	–	Cancer patients of different stages and subtypes were detected with 80% specificity	^99^
Intracran‐ial tumors	Detection and discrimination	Plasma	447 samples across 9 tumors types	cfMeDIP‐seq	The top 300 differentially methylated regions	This technology could accurately discriminate between common primary intracranial tumors	^100^
mPCa	Predict treatment	Plasma	97 control and 163 patients	MS‐ddPCR	DOCK2, HAPLN3 and FBXO30	ctDNA methylation of was detected in high‐volume (89.3%) mPCa and low‐volume (61.5%) mPCa	^101^
NMIBC	Monitoring of Recurrence	Urine	440 patients	Bladder EpiCheck	15 methylation markers	Sensitivity of 91.7% and specificity of 88%	^102^

Abbreviations: AUC, area under curve; BC, breast cancer; CRC, colorectal cancer; HCC, hepatocellular carcinoma; LC, lung cancer; MBS, methylation block score; mPCa, metastatic prostate cancer; MS‐ddPCR, methylation‐specific ddPCR; MYO1‐G, myosin IG; NMIBC, nonmuscle‐invasive bladder cancer; OPC, oropharyngeal cancer; Q‐MSP, quantitative methylation‐specific PCR.

The ctDNA methylation detection method has great potential in early cancer detection and recurrence monitoring, but its widespread application still faces multiple technical challenges. Specifically, these challenges include DNA fragmentation and loss, which are particularly evident in the key step of methylation analysis and may affect the accuracy of the results; sensitivity and specificity issues, especially in the early stages of cancer, where ctDNA concentrations are low, increasing the detection difficulty, and nontumor‐derived cfDNA may also interfere with the detection; limitations in low methylation detection, some methods cannot accurately determine low methylation, limiting their application; tissue specificity, the differences in methylation patterns between different tumor types and tissues make it difficult to develop universal detection methods; background methylation noise, which may interfere with detection results; the lack of standardization in detection methods, there is currently no uniform standard to evaluate the performance of different methods; sample handling and storage issues, which have a significant impact on the results; cost and efficiency considerations, PCR‐based methods are less expensive but have limited detection sites, while NGS‐based methods are expensive and time‐consuming; the need for personalized detection, which requires more resources and time for preliminary research and validation; and the challenge of rapid technological updates, clinical laboratories need to keep up and validate new technologies. In response to these challenges, researchers are constantly striving to improve and optimize ctDNA methylation detection methods. For example, they are developing more sensitive detection technologies, optimizing sample handling and storage conditions, standardizing operating procedures, and exploring personalized detection protocols to enhance the accuracy and reliability of detection. Additionally, with the advancement of big data and artificial intelligence technologies, it may be possible to automatically analyze and interpret methylation data using machine learning algorithms, further improving detection efficiency and accuracy.

## 3 CHALLENGES OF CTDNA METHYLATION

### 3.1 Challenges and coping strategies

Although liquid biopsy technology shows great potential in cancer management, many challenges still need to be overcome before it can be widely used in the clinic. These challenges include quality control of sample collection, processing, and preservation, as well as standardization and refinement of detection techniques.^4^ The main challenges of ctDNA methylation testing as a biomarker in liquid tumor biopsies are summarized as follows. *Sensitivity and specificity issues*: ctDNA content in the blood is extremely low, especially in the early stages of the tumor, and the detection sensitivity is difficult to improve. At the same time, the contamination of normal cell DNA also affects the specificity. Differences in sample collection and processing: For different types and different periods of tumors, there is no unified standard process for sample collection, ctDNA extraction and amplification, which affects the consistency and repeatability of detection results. *Tumor heterogeneity*: There is heterogeneity between tumor tissue and ctDNA, and between primary tumor and metastatic tumor, resulting in inconsistent detection results. *Technical cost and accessibility limitations*: The high cost of highly sensitive methylation sequencing technology limits its widespread clinical utilization. In view of the above challenges, researchers are actively exploring and implementing a series of solutions. In terms of sensitivity and specificity, the introduction of more advanced sequencing techniques and noise reduction methods is expected to significantly improve the accuracy and reliability of ctDNA methylation detection. In terms of sample processing and standardization, the development of a unified sample collection and processing protocol, combined with automated instruments and equipment, will help improve the stability and repeatability of test results. Aiming at the challenge of tumor heterogeneity, the application of multiomics combined analysis and big data technology will provide strong support for more comprehensive analysis of tumor heterogeneity. Finally, in terms of technology cost and accessibility, by optimizing sequencing technology and data processing processes, and developing portable, rapid detection equipment, it is expected to reduce detection costs and improve the popularity and accessibility of technology.

Sensitivity and specificity are the most critical and challenging problems in ctDNA methylation detection. Solving this problem requires not only highly sensitive sequencing techniques, but also a combination of advanced bioinformatics analysis and machine learning algorithms to distinguish between tumor‐specific methylation signals and background noise. The use of sequencing technologies such as WGBS and RRBS, combined with advanced enrichment and noise reduction methods such as cfDNA–RBS technology, can significantly improve the detection sensitivity and specificity. At the same time, the development of a highly sensitive targeted sequencing platform, combined with multimethylation PCR and noise reduction technology, and the construction of a scoring model based on machine learning algorithms, can accurately classify and evaluate methylation signals, and further improve the accuracy of detection results.

### 3.2 Improvements in traditional methylation detection techniques

Methylation detection technologies, pivotal in understanding epigenetic modifications, can be categorized based on genome coverage into three main types: WGBS, RRBS, and methylation capture technology. WGBS offers comprehensive coverage of all methylation sites and, despite its high cost, is unparalleled in detecting low‐frequency methylation signals when combined with targeted approaches. RRBS, while being the most cost‐effective among NGS technologies, suffers from less stable sequencing data due to its reliance on MspI cleavage, covering approximately 10% of methylation sites. Methylation capture technology, positioned cost‐wise between WGBS and RRBS, employs hybridization probes to enrich methylation‐specific fragments, resulting in more stable data than RRBS. WGBS stands out as the optimal screening method, capable of assessing all CpG sites. We have advanced the ctDNA–WGBS technique, proving its efficacy in analyzing comprehensive methylation patterns across the entire genome with minimal ctDNA quantities.^50^ Remarkably, this method requires only 200 µL of plasma, significantly less than the traditional 5–20 mL. As detailed in Figure 3C, we streamlined the library preparation process by integrating end repair, dA tailing, adapter ligation, and bisulfite conversion into a single tube, enhancing efficiency. Additionally, we replaced agarose gel capture with bead‐based methods to improve the recovery ratio. This refined approach allows for library preparation from as little as 1 ng of input, ensures unbiased genome‐wide coverage, and incorporates advanced computational strategies to filter out noise from low recurrent fragments and nontumor‐derived ctDNA. Consequently, it facilitates highly specific and sensitive detection of early‐stage BC across various patient cohorts and distinguishes between different cancer molecular subtypes. Despite its widespread use, bisulfite sequencing, the gold standard for DNA methylation detection, faces significant challenges, particularly with limited samples like cfDNA in blood. Bisulfite treatment can degrade up to 84−96% of DNA, complicating the analysis of scarce samples. Moreover, bisulfite sequencing indirectly detects 5mC and 5hmC, converting unmodified cytosines to uracil, which diminishes sequence complexity, degrades sequencing quality, and hampers efficient targeting.

### 3.3 Improvement of bisulfite‐free technology

In response to the limitations of traditional sequencing methods, several bisulfite‐free technologies have been developed. Shen et al.^103^ introduced cfMeDIP‐seq, a novel approach that combines immunoprecipitation with high‐throughput sequencing to specifically enrich methylated DNA fragments in cfDNA, thereby enhancing detection efficiency. Similarly, researchers at the University of Pennsylvania have developed APOBEC‐coupled epigenetic sequencing. This technique replaces the chemical transformation of bisulfite with an enzymatic process, using the APOBEC deaminase enzyme to effectively distinguish between cytosine states without causing DNA damage.^104^ Song et al.^105^ discovered a sulfite‐free, base‐resolution sequencing method known as TET (ten‐eleven translocation)‐assisted pyridine borane sequencing. This method employs the TET enzyme to convert 5mC and 5hmC into a third modification, which is then chemically converted to thymine for direct sequencing. Yuming Lu's team has developed a methodology for the direct detection of 5mC using single molecule real‐time (SMRT) sequencing, providing a comprehensive examination of the kinetic signals of DNA polymerase and the sequence context of each nucleotide. This approach achieved a sensitivity and specificity of 90 and 94%, respectively, with a 99% correlation to overall methylation levels compared with sulfite sequencing.^106^ Nanopore sequencing offers direct detection of methylated cytosines without the need for bisulfite conversion. Adaptive sampling, a method that enriches regions of interest by removing off‐target regions during sequencing, has been utilized by Danny et al.^107^ in conjunction with the Oxford Nanopore platform for targeted long‐read sequencing. This approach allows for the identification of pathogenic substitutions, structural variants, and methylation differences using a single data source. However, the requirement for unamplified genomic DNA may limit the application of single‐molecule approaches like SMRT‐seq and Nanopore sequencing in rare clinical samples. To address this, Li et al.^108^ developed NT‐seq, a method that sequences multiple DNA methylations based on chemical transformation, enabling the detection of various DNA epimethylations at the genome‐wide level. Most recently, Wang et al.^109^ introduced direct methylation sequencing (DM‐Seq), an all‐enzymatic method that uses nanogram quantities of DNA for single‐base resolution analysis of 5mC. DM‐Seq offers a nondestructive, reliable, and direct detection method for 5mC, overcoming the traditional challenges of distinguishing between 5mC and 5hmC.

### 3.4 Optimization algorithms and models

GRAIL's cfDNA‐targeted methylation liquid biopsy assay represents a significant advancement in cancer detection, capable of identifying and localizing over 50 types of cancer, including those with high mortality rates and lacking screening guidelines. This assay achieves a remarkable specificity of 99.3% and can accurately identify the tissue source of the cancer signal with a 93% accuracy rate when a cancer signal is detected.^110^ However, the predictive values calculated by GRAIL currently rely on incidence rates rather than prevalence rates, which may not be the most logical approach. Fiala and Diamandis^111^ have critiqued the company's study for comparing asymptomatic subjects with known cancer patients, arguing that this inflates the test's positive predictive value and does not accurately reflect its use in screening the general population. To address the high costs associated with high‐throughput sequencing, Li et al.^112^ proposed DISMIR, a novel method incorporating a “switching region” to identify cancer‐specific differentially methylated regions. DISMIR, leveraging deep learning models, has shown high precision and robustness in detecting HCC from plasma cfDNA WGBS data, even at ultra‐low sequencing depths. Its insensitivity to individual CpG site methylation status changes suggests resistance to WGBS technical noise, positioning DISMIR as a promising tool for early cancer detection.

In the Circulating Cell‐free Genome Atlas Substudy,^113^ researchers evaluated 10 sequencing analysis technologies targeting cfDNA for early tumor screening. Findings indicated that cfDNA methylation is the most effective marker for predicting cancer signal sources, with genome‐wide methylation classifiers exhibiting the highest sensitivity. This approach has demonstrated the capability to detect common cancer signals across more than 50 cancer types with 99.5% specificity and accurately predict cancer origins. Wang and colleagues^114^ introduced mutation capsule plus (MCP) technology, enhancing liquid biopsy assays for HCC detection compared with current assays or evaluating methylation or mutation markers alone. MCP technology holds promise for discovering and validating noninvasive cancer detection biomarkers.

Chemi et al.^30^ developed a methodology for genome‐wide DNA methylation analysis applicable to patient‐derived models and cfDNA, improving SCLC detection sensitivity and distinguishing between different progression stages. David et al.’s^115^ “HCC‐detect” classifier, based on four CpG sites, identified 98% of HCC samples with 100% specificity, differentiating HCC from various liver conditions. Their epiLiver assay, utilizing a focused multiplexed high‐throughput next‐generation sulfite sequencing approach, classified HCC patients with 84.5% sensitivity and 95% specificity. Building on a comprehensive tissue methylation atlas from 521 noncancerous tissue samples across 29 major human tissues, Li et al.^116^ developed cfSort, a novel deep‐learning model for precise tissue identification within cfDNA. cfSort outperformed existing methods in benchmark data, enhancing tissue deconvolution performance in cfDNA for disease detection and therapy monitoring. However, accurately quantifying tissue‐derived cfDNA remains a challenge due to limited knowledge on tissue methylation and reliance on unsupervised techniques, highlighting the need for further research in this area.

In summary, as an important biomarker in tumor liquid biopsy, ctDNA methylation detection faces many challenges, but through continuous technological innovation and optimization, it is expected to become an important tool for early tumor screening and diagnosis in the future.

## 4 CLINICAL APPLICATIONS OF CTDNA METHYLATION IN PRECISION ONCOLOGY

**FIGURE 4:**
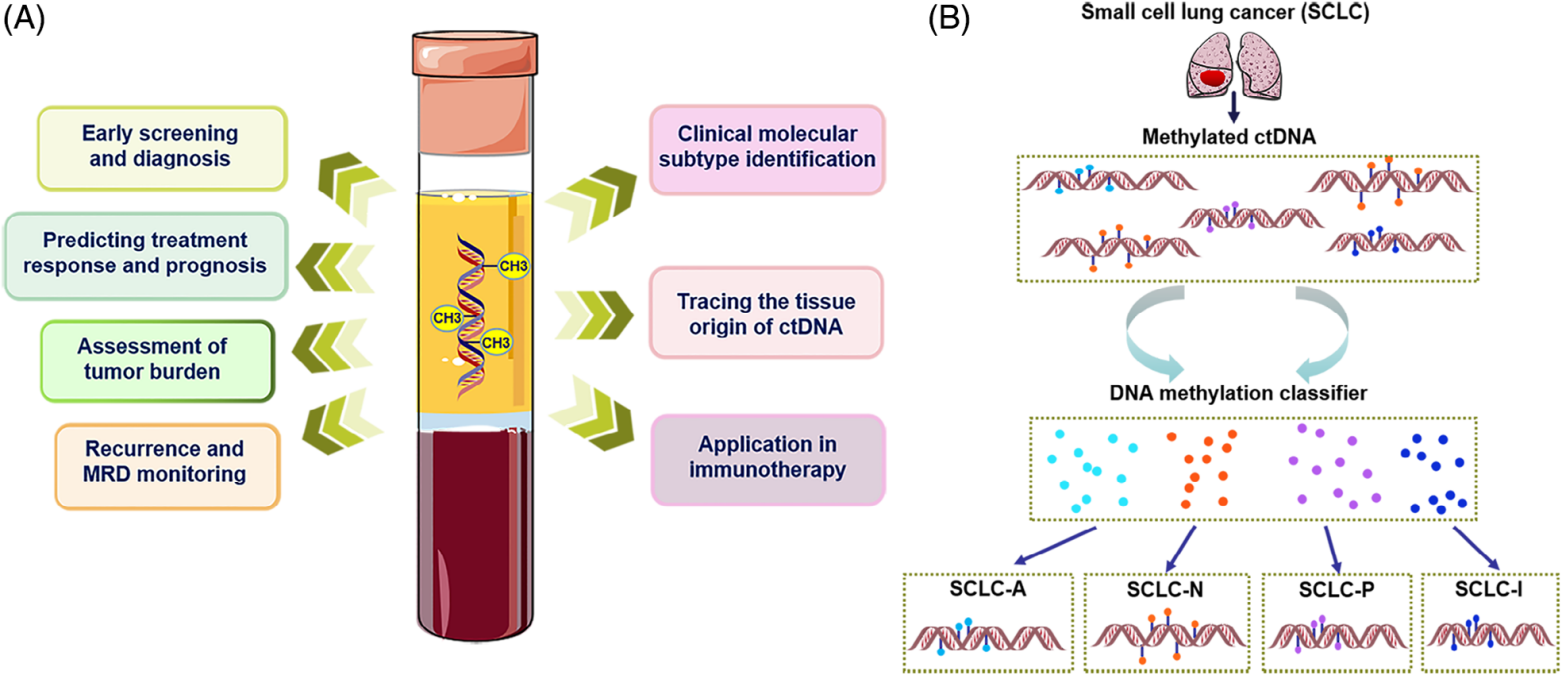
Clinical application of ctDNA methylation in precision oncology. (A) Collection tube: ctDNA derived from apoptotic or necrotic tumor cells can be extracted from body fluids. The analysis of ctDNA enables the detection of alterations in DNA methylation patterns. Block diagram: shows the potential clinical applications of ctDNA methylation analysis in disease management throughout the progression of the condition. (B) Taking the latest clinical molecular subtype identification as an example, DNA methylation‐based classifier was used to classify SCLC based on plasma‐derived methylation data. Different SCLC subtypes have different DNA methylation, and the evolution of SCLC subtypes can be tracked by longitudinal evaluation through liquid biopsy.

The clinical application of ctDNA methylation in precision oncology, as depicted in Figure 4, underscores its pivotal role in cancer screening, detection, and the monitoring of MRD and recurrence. ctDNA methylation‐targeted sequencing has been shown to outperform FISH and DNA mutation analysis in tumor detection, recurrence, and prognosis assessment.^117^ This approach enables the early detection of cancer, facilitating timely intervention and treatment, and allows for the identification of high‐risk patients for stratified treatment, with multidrug resistance serving as a predictor for recurrence.^1^ Recent studies have classified SCLC into subgroups based on biological characteristics, aiding in the selection of precision treatment plans.^118^ The inflammatory subtype (SCLC‐I), for instance, is characterized by higher levels of programmed cell death‐Ligand 1 (PD‐L1) transcription, interferon signaling, and epithelial‐mesenchymal transition.^32^ Heeke et al.^31^ reported a robust detection method capable of distinguishing SCLC subtypes through both tissue and liquid biopsies by analyzing gene expression and DNA methylation patterns. This method facilitates the tracking of SCLC subtype evolution via longitudinal liquid biopsy evaluations (Figure 4B). SCLC classification based on plasma‐sourced methylation data achieved a 100% accuracy using the gene ratio classifier (SCLC‐GRC) based on tissue‐sourced mRNA data, and a 93.3% accuracy using the DNA methylation‐based classifier (SCLC‐DMC) based on tissue‐sourced methylation data. This machine learning approach, utilizing DNA methylation data applicable to cfDNA detected by liquid biopsy, lays a theoretical foundation for the discovery of predictive and prognostic biomarkers and the advancement of clinical precision treatment. Furthermore, the study highlighted the prognostic value of methylated BCAT1/IKZF1 levels in plasma for assessing the risk of CRC recurrence, with the application of the upper reference limit (URL) assay enhancing specificity.^119^ The correlation between ctDNA changes and treatment outcomes offers a means to evaluate treatment effectiveness, identify response or progression, and adjust treatment strategies accordingly. Recent research has demonstrated that ctDNA methylation patterns can serve as biomarkers for diagnosing and predicting the prognosis of thyroid cancer,^120^ emphasizing the importance of determining the tissue source of cfDNA due to its significant biological implications and diagnostic value. The development of a comprehensive high‐resolution tissue methylation map and the first supervised tissue deconvolution method based on cfDNA, cfSort, enables sensitive and robust quantification of tissue fractions in cfDNA, enhancing cfDNA‐based disease detection and longitudinal treatment monitoring.^116^ The study also revealed that MYO1‐G methylation levels correlate with tumor burden and treatment response, with postradical surgery patients showing significantly lower methylation rates compared with preoperative levels, and patients with progressive disease exhibiting higher methylation rates than those with stable disease or complete/partial remission.^91^ These findings underscore the clinical potential of analyzing circulating DNA quantitatively and qualitatively to improve early detection and treatment outcomes for both early‐stage and advanced HCC cases. In BC diagnostics, an efficient method, BC‐mqmsPCR, was developed using multiplex quantitative PCR on four methylation loci, showing high diagnostic accuracy with an AUC of 0.913 for distinguishing BC from other cancers, and an AUC of 0.945 with a sensitivity of 93.2% for diagnosing early‐stage BC.^35^ Similarly, a CRC diagnostic model based on DNA methylation sites and multiple methylation‐specific quantitative PCR techniques demonstrated an AUC of 0.93, a sensitivity of 77.14%, and a specificity of 92.31%,^36^ highlighting the utility of DNA methylation analysis in cancer diagnostics. Dai et al.^121^ have discovered new diagnostic and prognostic biomarkers for glioma patients through methylation analysis of ctDNA in CSF, which can help precision medicine for glioma patients.

### 4.1 Early screening and diagnosis

In recent years, the exploration of ctDNA as a diagnostic tool for various cancers has garnered significant research interest, as outlined in Table 2. The application of ctDNA methylation is emerging as a promising strategy for the early detection of cancer. Cancer‐specific methylation patterns, which appear at the early stages of tumor development and exhibit remarkable stability, provide a highly accurate signal for precise analysis.^122^ Klein et al.^123^ reported a large‐scale clinical validation of a methylation‐based classification in 4077 participants, achieving a specificity rate of 99.5% for cancer detection. This underscores the potential of methylation‐based ctDNA analysis to enhance early‐stage cancer screening across various types. Research focused on early CRC detection through ctDNA methylation, particularly SEPT9 hypermethylation, has demonstrated high sensitivity and specificity.^124^ This approach has shown promise in surpassing traditional screening methods, such as endoscopy, especially in high‐risk groups for CRC.^125^ The diagnostic efficacy of CRC has been significantly enhanced by combining methylation markers with current markers like carcinoembryonic antigen (CEA) and fecal immunochemical test, achieving higher sensitivity and improved AUC values.^126^ Furthermore, a study by Brenne et al.^127^ indicates that known methylation markers of CRC detected by liquid biopsy can be detected up to 2 years before clinical diagnosis, suggesting its potential for early screening of CRC. Yu et al.^128^ developed a highly sensitive screening method for esophageal cancer using five candidate markers with significant hypermethylation, suggesting DNA methylation‐based molecular detection as a novel approach for esophageal adenocarcinoma screening. Similarly, ctDNA methylation detection has shown high sensitivity in identifying early‐stage LC, indicating its potential as a method for early diagnosis and screening.^64^, ^129^ Liang et al.’s^130^ model, PulmoSeek, which incorporates 100 methylation features, demonstrated high sensitivity in detecting early‐stage LC, outperforming existing diagnostic models and positron emission tomography‐computed tomography in precision. In addition, Bu et al.^131^ evaluated the application of Septin9 gene methylation in the diagnosis of cervical cancer and prediction of pelvic lymph node metastasis, demonstrating its potential as a novel biomarker. Lung EpiCheck, a novel blood test based on six methylation markers, has shown high detection rates for early non‐small cell lung cancer (NSCLC) and SCLC in high‐risk populations.^132^ Additionally, the detection of NSCLC‐specific ctDNA in urine through methylation analysis of CDO1 and SOX17 gene markers has been explored, indicating the feasibility of this noninvasive method for NSCLC detection.^133^ In HCC research, ctDNA detection precedes imaging abnormalities and elevated alpha‐fetoprotein (AFP) levels,^134^ with hypomethylation near hepatitis B virus integration sites serving as an effective early screening method.^135^ The hypermethylation status of additional genes, such as the CTCFL promoter and UBE2Q1 in ctDNA, is associated with HCC diagnosis and patient monitoring.^136^, ^137^ The detection of ctDNA methylation levels in serum/plasma samples from OC patients aids in early detection and guides individualized treatment.^69^, ^138^, ^139^ Promoter region methylation inactivating tumor suppressor genes is a significant factor in OC development,^138^, ^140^ warranting further investigation. Li et al.^42^ analyzed DNA methylation kinetics in consecutive CSF samples, demonstrating the high sensitivity and clinical potential of ctDNA methylation labeling for monitoring MB illness status. In conclusion, ctDNA methylation marker detection offers a noninvasive and effective means to improve adherence and participation in cancer screening, highlighting its significant potential in precision oncology.

**TABLE 2:** Application of ctDNA methylation markers in early diagnosis and screening of cancer.

Cancer type	Application	Sample type	Sample size	Methodology	ctDNA methylation biomarkers	Main findings	References
ENKTL	Diagnostic prediction	Plasma	594 patients and 734 healthy subject	Variables were screened using LASSO logistic regression	7‐methylation marker	The sensitivity of the training set was 94.26%, the specificity was 97.57%, and the AUC was 0.989.	^26^
BC	Early diagnosis	Serum	749 (women with BC, benign disease, and normal control)	High‐throughput MethyLight	Six‐gene panel	82.4% sensitivity and 78.1% specificity	^70^
BC	Early detection	Serum	101 cases of primary BC and 58 cases of metastatic BC and 87 healthy controls	OS‐MSP	GSTP1, RASSF1A, and RARb2	When OS‐MSP is combined with CEA and/or CA15‐3 tests in metastatic BC, sensitivity increases to 78%.	^71^
CRC	Detection	Plasma	193 patients and 102 normal controls	MSP	Seven hypermethylated promoter regions	Sensitivity and specificity of 90.7% and 72.5%	^83^
CRC	Diagnose	Blood	272 patients and 402 normal samples.	ddPCR.	MYO1‐G	84.3% sensitivity and 94.5% specificity, AUC = 0.94	^91^
Multicancer	Early detection	Plasma	2823 with cancer and 1254 normal samples.	Methylation‐based MCED test and WGBS	–	Specificity was 99.5%, TOO was predicted with 88.7% accuracy.	^123^
CRC	Diagnose	Plasma	104 cases of CRC and 130 cases of colorectal polyps, 60 normal controls	RT‐PCR	Septin9, SDC2, and BCAT1	The sensitivity, specificity, and AUC were 83.7, 93.9, and 0.908, respectively.	^126^
NSCLC	Early detection	Serum	43 stage IA patients and 42 control	Q‐MSP	6 methylation markers	Sensitivity over 90%	^129^
LC	Diagnose	Plasma	529 patient samples (116 benign and 413 malignant)	PulmoSeek	100 methylation features	The AUC of PulmoSeek is 0.843 [0.769–0.918] and the sensitivity is 0.990 [0.610–1.000].	^130^
Nonmetastatic NSCLC	Early detection	Urine	46 surgical patients and normal controls	qMSP	CDO1 and SOX17	CDO1 And SOX17 Methylation Levels Were Significantly Increased In Patients With An AUC Value Of 0.71.	^133^
BCa	Early detection	Urine	157 patients and 339 control	MSP	TWIST1 and NID2	Sensitivity of 90% for the presence of primary BLCA	^141^
BCa	Diagnose	Urine	86 patients and 30 normal control	UroMark	150 CpG loci biomarker panel	Sensitivity and specificity of 98% and 97%	^142^
LC	Early diagnosis	Blood	70 patients and 100 healthy control	RT‐PCR	SEPT9	44.3% patients were hypermethylated, in healthy controls (specificity 96%).	^143^
CRC	Early screening	Plasma	53 patients and 1457 normal control	Epi proColon Assay	SEPT9	48.2% sensitivity and 91.5% specificity	^144^
CRC	Early diagnosis	Blood	801 patients and 1021 healthy control	cd‐score‐based classifier	9 methylation markers	Sensitivity of 87.9% and specificity of 89.6%	^145^
CRC	Early screening	Plasma	369 CRC patients, 274 patients with other diseases and 490 normal samples.	RT‐qPCR; Epi proColon 2.0 CE	SEPT9	75.1% sensitivity and 95.1% specificity	^146^
HCC	Diagnose	Plasma	1098 patients and 835 healthy control	bis‐DNA captured by molecular‐inversion (padlock) probes	10 methylation markers	The sensitivity in the training dataset is 85.7%, with a specificity of 94.3%; in the validation dataset, the sensitivity to liver cancer is 83.3%, with a specificity of 90.5%.	^147^
CRC	Detection	Plasma	43 patients and 42 healthy controls	Dual‐strand digital PCR; TriMeth	C9orf50, CLIP4, and KCNQ5	The TriMeth test demonstrates excellent sensitivity (85%) and specificity (99%).	^148^
CRC	Screening	Plasma	A total of 2105 volunteers, of which 26 samples underwent cancer surgery	Methylation specific real‐time qPCR	BCAT1 and IKZF1	Sensitivity and specificity of 66 and 94%.	^149^
HCC	Early detection	Plasma	146 patients and 98 normal control	TELQAS	A six‐marker panel of methylated DNA	Sensitivity of 95% and specificity of 92%	^150^

Abbreviations: BCa, bladder cancer; ENKTL, extranodal natural killer/T cell lymphoma; Epi proColon, Epigenomics; LASSO, least absolute shrinkage and selection operator; MCED, multicancer early detection; NSCLC, non‐small cell lung cancer; RT‐qPCR, real‐time quantitative polymerase chain reaction; TELQAS, target enrichment long‐probe quantitative amplified signal.

### 4.2 Monitoring for recurrence and MRD

Recent advancements have highlighted the significant role of ctDNA methylation in cancer surveillance, particularly in detecting MRD and tumor recurrence. Michail et al.^151^ first introduced the concept of “ctDNA recurrence,” underscoring ctDNA's critical role in monitoring cancer. Postoperative or after curative therapy, the presence of MRD is a primary cause of tumor recurrence. Numerous studies have demonstrated the utility of ctDNA methylation markers in monitoring tumor recurrence and MRD (Table 3), offering advantages such as rapid turnaround time, low initial cost, and applicability in cases where tumor tissue is unavailable or inaccessible.^38^ For instance, the detection of methylated BCAT1 and IKZF1 in blood has shown potential in identifying residual disease posttreatment, suggesting a need for treatment modification in advanced‐stage illness.^47^, ^152^, ^153^ This is further supported by Wang et al.,^25^ who developed a 5‐marker ctDNA methylation model with 87.5% sensitivity and 94.12% specificity for predicting tumor recurrence in CRC patients. This model allows for earlier CRC recurrence detection than imaging techniques and serum CEA measurement, providing a basis for early treatment adjustment. Pedersen et al.^154^ established a reference level for plasma BCAT1 and IKZF1 methylation markers, enhancing specificity and clinical accuracy for recurrence detection. Al Naji et al.^155^ evaluated the predictive value of BCAT1/IKZF1 ctDNA methylation for CRC recurrence and found that its combination with clinical prognostic factors significantly improves prediction accuracy. Similarly, elevated levels of methylated SEPT9 ctDNA have been identified as a significant biomarker for CRC recurrence,^6^ offering superior potential over CEA.^156^ Jin et al.’s^82^ mqMSP assay targeting the SEPT9 gene predicted CRC recurrence within 2 weeks of surgery, outperforming traditional CEA markers. A prospective clinical trial measuring ctDNA methylation levels of WIF1 and NPY pre‐ and postsurgery in stage II–III CRC patients found a higher 2‐year DFS rate in ctDNA‐negative patients,^157^ indicating the potential of ctDNA methylation analysis in predicting postoperative MRD. Slater et al.^158^ conducted a study and proposed that longitudinal sampling of MRD assessment can predict recurrence in stage I–III CRC, providing a basis for individualized treatment. Additionally, hypermethylation in ctDNA of genes like RASSF1A, COX2, and APC has been linked to increased tumor recurrence and poorer survival in HCC patients.^159^ Research on OC patients has shown that DNA methylation in genes such as SFRP1, SFRP2, SOX1, and LMX1A is associated with a higher risk of recurrence and poorer overall survival (OS) outcomes.^160^ Furthermore, ctDNA methylation detection offers promising avenues for identifying MRD and monitoring tumor progression. Parikh et al.^18^ provided an analytical method using a focused gene panel and methylation markers to assess MRD, eliminating the need for resected tumor tissue. This MRD assay, utilizing plasma alone, demonstrated high specificity and sensitivity in detecting recurrence, with further enhancements achieved by incorporating epigenomic markers and analyzing serial longitudinal samples.^18^ The ongoing NCT04068103 trial employs this assay to inform treatment decisions in stage IIA colon cancer,^38^ showcasing the potential of a tumor‐blind methodology in ctDNA‐based MRD detection. These advancements highlight the dynamic monitoring of tumors using ctDNA methylation as an effective strategy for early detection of tumor recurrence and facilitating clinical decision‐making.

**TABLE 3:** Application of ctDNA methylation markers in recurrence and MRD monitoring of cancer.

Cancer type	Application	Sample type	Sample size	Methodology	ctDNA methylation biomarkers	Main findings	References
CRC	Predict recurrence	Plasma	252 plasma samples from 103 posttreatment patients	Guardant Reveal test; MRD assay	Integrating epigenomic signatures	The specificity is 100% and the sensitivity is 91% for recurrence.	^18^
CRC	Monitoring of recurrence	Plasma	Different stages of 130 patients	High‐throughput targeted DNA sequencing and random forest method.	SFMBT2, SGCG, ZNF568, ZNF671, and ZNF132)	The sensitivity and specificity were 87.5 and 94.12%, respectively.	^25^
CRC	Predict recurrence	Plasma	479 patients	Triplex RT‐qPCR assay	BCAT1 and IKZF1	Positive ctDNA after surgery was linked to a higher risk of recurrence (hazard ratio 3.8, *p* = 0.004).	^43^
CRC	Predict recurrence	Plasma	322 patients for final analysis	Tests were performed with COLVERA and CEA.	BCAT1and IKZF1	The sensitivity and specificity were 63 and 91.5%, respectively.	^47^
CRLM	Monitoring of recurrence	Plasma	84 patients (60 patients with recurrence and 24 patients without recurrence)	Tumor‐agnostic methylation multiplex ddPCR “TriMeth”	TriMeth	The sensitivity is 90.0% and the specificity is 83.3% for recurrence.	^77^
CRC	Monitoring of recurrence	Plasma	182 samples were obtained from 82 CRC patients	mqMSP	10 different methylation markers	ctDNA predicted recurrence 8.0 months earlier than radiographic examination.	^82^
NMIBC	Monitoring of recurrence	Urine	440 patients	Bladder EpiCheck	15 methylation markers	The sensitivity was 67% for NMIBC recurrence, 91.7% for MIBC recurrence, and the specificity was 88%.	^102^
CRC	Predict recurrence	Plasma	142 CRC stage I–III cases	RT‐PCR; URL ≥ 0.07%; log‐rank (Mantel–Cox)	BCAT1 and IKZF1	The 3‐year RFS rate of BCAT1/IKZF1 negative patients (83.3%) was higher than that of BCAT1/IKZF1 positive patients (56.5%).	^119^
CRC	Detection of recurrence	Plasma	857 in reference population and 549 in posttreatment population	The methylation level of 0.07% was used as the URL for quantitative determination	BCAT1 and IKZF1	The sensitivity and specificity of recurrence detection were 63.6 and 97.7%, respectively.	^154^
CRC	Predict recurrence	Plasma	805 patients before and after surgery	ddPCR	WIF1 and NPY	2‐year DFS was higher in ctDNA‐negative patients (82%) than in ctDNA‐positive patients (64%).	^157^
BC	Monitoring of recurrence	Plasma	87 patients with stage II–III primary BC	OS‐MSP	RASSF1A	Postoperative methylated ctDNA was significantly correlated with residual tumor burden (*p* = 0.008).	^161^
CRC	Monitoring of recurrence	Serum	20 healthy donors and 21 sporadic CRC patients	Q‐MSP	P16INK4a	The methylation level exhibited a decline in all patients within a fortnight postsurgery, followed by a notable surge upon recurrence.	^162^

Abbreviations: COLVERA, a qualitative blood test that detects methylated BCAT1 and IKZF1 in cfDNA; CRLM, colorectal liver metastases; DFS, disease‐free survival; RFS, recurrence‐free survival.

### 4.3 Predicting treatment response and prognosis

The determination of prognosis is significantly influenced by ctDNA methylation models, which utilize blood DNA methylation to develop prognostic models. This approach enables clinicians to quickly identify high‐risk individuals, providing timely and effective interventions for those at risk and offering less‐intensive treatments or follow‐up for those not at high risk. By doing so, it avoids the widespread use of traumatic therapies that can negatively affect patients and waste medical resources. Extensive research on ctDNA's ability to predict cancer prognosis (Table 4), including in OC, CRC, and prostate cancer, has yielded improved predictive outcomes.^8^, ^163^, ^164^, ^165^ Studies have shown a correlation between ctDNA dynamics and treatment response,^147^, ^166^ with Boeckx et al.^167^ demonstrating that changes in ctDNA concentrations can reflect individual treatment responses by combining mutated and methylated ctDNA biomarkers. For instance, a study found significant reductions in ctDNA methylation levels of HPP1 after induction chemotherapy,^168^ suggesting its potential as a prognostic marker. The challenge of preventing metastatic spread in locally advanced rectal cancer highlights the need for effective prognostic factors for pretreatment metastatic progression. In this context, ctDNA quantification by assessing BCAT1 and IKZF1 methylation levels has shown that posttreatment ctDNA detection is associated with disease progression,^169^ offering valuable insights for clinical decision‐making regarding further treatment or monitoring. Postoperative increases in ctDNA methylation levels are associated with poor tumor prognosis. For example, SEPT9 methylation levels in CRC patients dropped significantly after surgery,^170^ while postoperative detection of plasma SEPT9 hypermethylation was linked to higher mortality rates.^171^ Similarly, methylation status has been identified as a valuable biomarker for predicting NSCLC prognosis,^172^, ^173^ with studies showing that specific methylation patterns are associated with unfavorable outcomes.^174^, ^175^ In high‐grade serous ovarian cancer (HGSOC), the prognostic significance of methylation in genes such as SOX17,^176^ CST6,^177^ BRMS1,^178^ RASSFIA,^69^ and ESR1^179^ in plasma ctDNA has been demonstrated. Additionally, GSTP1 promoter methylation in ctDNA has been analyzed^180^, ^181^ and has been proven useful for prognosticating metastatic castration‐resistant prostate cancer.^182^ Tian et al.^26^ developed a cmp score based on methylated ctDNA composition to predict ENKTL prognosis, highlighting the need for further clinical trials to validate its practical application. In rhabdomyosarcoma, RASSF1A‐M methylation in cfDNA was significantly associated with poor prognosis.^183^ While the prognostic value of ctDNA methylation patterns has been observed across various cancers, the refinement and validation of methylation markers for prognostic assessment continue to be crucial areas of research.

**TABLE 4:** Application of ctDNA methylation markers in predicting cancer treatment response, prognosis, and assessing of tumor burden.

Cancer type	Application	Sample type	Sample size	Methodology	ctDNA methylation biomarkers	Main findings	References
HGSOC	Assessment of prognostic	Plasma	133 plasma samples from patients	Real‐Time MSP	BRCA1, CST6, MGMT, RASSF10, SLFN11, and USP44	SLFN11 DNA methylation was significantly associated with poorer PFS (*p* = 0.045).	^8^
CRC	Assessment of tumor burden	Plasma	Different stages of 130 patients	Ultrasensitive high‐throughput targeted DNA sequencing and random forest method.	Five‐methylation‐marker model(SFMBT2, SGCG, ZNF568, ZNF671, and ZNF132)	The sensitivity and specificity of the five‐methylation markers model were 87.5 and 94.12%, respectively, which could represent the potential metastatic tumor burden of CRC patients.	^25^
ENKTL	Prognostic prediction	Plasma	594 patients and 734 healthy subject	LASSO‐Cox methods	7‐methylation marker	Compared with cmp score ≤−23.1, patients with cmp score >−23.1 had a higher risk of death and progression, and a significantly reduced PFS.	^26^
OC	Prognostic prediction	Plasma	23 patients	HOXA9 meth‐ctDNA	HOXA9	Patients with increased levels of HOXA9 methylation in ctDNA had lower PFS and OS compared with patients with stable or decreased levels (1.4 months and 4.3 months vs. 7.8 months and 12 months).	^40^
mPCa	Predict treatment	Plasma	167 cancer patients, 61 benign patients and 36 healthy controls	MS‐ddPCR	DOCK2/HAPLN3/FBXO30	Methylation of ctDNA is significantly associated with a shortened time to cancer progression, independent of tumor volume.	^101^
mPCa	Assessment of tumor burden	Plasma	167 cancer patients, 61 benign patients and 36 healthy controls	MS‐ddPCR	DOCK2/HAPLN3/FBXO30	The level of ctDNA methylation in mPCa patients with high tumor volume was significantly higher than that in those with low tumor volume (*p* < 0.001).	^101^
GC	Assessment of prognostic	Plasma	107 patients(99 GC patients and 8 benign disease patients)	qPCR;HELP	LINE‐1	Postoperative LINE concentration was negatively correlated with RFS and OS (*p* = 0.009, *p* = 0.04).	^125^
HCC	Prognostic prediction	Blood	1098 patients and 835 healthy controls	bisulfite sequencing	8‐Marker prognostic model	The sensitivity was 90.5% and the specificity was 83.3%.	^147^
CRC	Assessment of tumor burden	Plasma	306 patients	Multiplex RT‐qPCR	BCAT1 and IKZF1	ctDNA levels increased with the progression of staging and were positively correlated with tumor diameter (*p* < 0.001) and volume (*p* < 0.01). After treatment, ctDNA levels decreased in 98% of patients.	^169^
CRC	Prognostic prediction	Plasma	120 patients	Epi proColon 2.0 CE	SEPT9	The level of mSEPT9 decreased significantly after surgery, and 86.7% of patients could be evaluated by mSEPT9.	^171^
RMS	Prognostic prediction	Plasma	152 plasma samples from 65 patients	shWGS	RASSF1A	RASSF1A‐M is significantly associated with poor prognosis (*p* < 0.001).	^183^
BC	Prognostic prediction	Serum	336 primary invasive breast cancer patients	OS‐MSP	GSTP1, RASSF1A, and RARb2	Compared with unmethylated promoters, promoter methylation is correlated with significantly lower OS (95 vs. 78%)	^184^
BC	Monitor therapy	Plasma	20 patients and 20 healthy control	MethDet‐56	PROX, MDGI, PAX5, RARb2, ESR1 B	Compared with the healthy control group, the methylation level increased. After surgery, the methylation level decreased, and tamoxifen treatment altered the methylation of ESR1B.	^185^
CRC	Monitor therapy	Plasma	467 patients	MSP	HPP1	mHPP1 ctDNA could distinguish treatment responders from nonresponders.	^186^
HCC	Monitoring of prognostic	Plasma	109 patients and 41 normal controls	MSRE‐PCR	A set of four genetic markers	The AUC of the combination of four genes is 0.933, the sensitivity is 92.7%, and the specificity is 81.9%, and it could predict the prognosis.	^187^
OC	Prognostic prediction	Plasma	32 patients	ddPCR	HOXA9	The PFS of HOXA9 methylated ctDNA positive patients (5.1 months) was lower than that of negative patients (8.3 months).	^188^
CRC	Monitoring surgical	Plasma	187 cases providing peri‐diagnostic blood samples	RT‐MSP	BCAT1 and IKZF1	Among 47 patients with positive ctDNA, 35 (74.5%) became negative after tumor resection.	^189^
CRC	Prognostic prediction	Serum	146 patients	ddPCR	NPY	Patients with ctDNA methylation positivity have a significantly poorer 5‐year survival rate(47 vs. 69%).	^190^

Abbreviations: GC, gastric cancer; HGSOC, high‐grade serous ovarian cancer; MethDet‐56, methylation detection in 56 genes; NPY, neuropeptide Y; OC, ovarian cancer; OS, overall survival; PFS, progression‐free survival; RMS, rhabdomyosarcoma; shWGS, shallow whole genome sequencing.

### 4.4 Assessing tumor burden

The short half‐life of cfDNA directly correlates with tumor cell status, offering a unique opportunity for continuous monitoring of cancer burden during treatment. Recent studies have explored the utilization of ctDNA methylation markers for assessing tumor burden (Table 4). Melton et al.^191^ proposed a new statistical method that quantifies the cancer‐specific methylation patterns in cfDNA to estimate ctDNA abundance, which helps noninvasive assessment of tumor burden. Bjerre et al.^101^ detected a positive correlation between tumor burden and plasma methylated ctDNA levels, suggesting that methylated ctDNA could serve as a biomarker to identify patients with mPCa with high tumor volume, potentially guiding therapy decisions for patients at this stage.^101^ However, these findings are limited by the small number of mPCa patients in the study, necessitating further validation in a larger cohort. In another study, methylation mDETECT levels were shown to predict elevated prostate‐specific antigen (PSA) levels, indicating tumor burden in PSA‐negative tumors.^182^ In addition, a study by Angeli‐Pahim et al.^192^ indicates that quantifying methylation of ctDNA can effectively monitor changes in tumor burden in HCC patients without the need for tumor biopsy. Tran et al.^193^ analyzed 366 plasma ctDNA samples from 85 patients, using CT‐based three‐dimensional annotated tumor volumes to identify early‐stage NSCLC patients at risk for recurrence. This integrated imaging analysis could stratify risk groups for clinical outcomes. Symonds et al.’s^169^ research quantified ctDNA by assessing BCAT1 and IKZF1 methylation levels, demonstrating the potential of this approach to evaluate tumor burden and treatment response. In a study of 175 patients with pretreatment‐positive CRC ctDNA, levels increased with disease progression and were significantly correlated with tumor diameter and volume. Methylation levels of BCAT1 and IKZF1 DNA decreased substantially in 48 fully treated cases, with undetectable levels in the majority (87.5%) of patients. This analysis revealed a consistent trend of increasing ctDNA levels with tumor mass.^169^ To investigate the potential of using blood mSEPT9 to evaluate treatment response in CRC, Song et al.^171^ found that mSEPT9 levels were associated with tumor size, unlike CEA levels. This suggests that ctDNA methylation markers offer a promising approach for real‐time monitoring of tumor dynamics and assessing the effectiveness of treatment strategies.

### 4.5 Determine the source of the organization

Epigenetic biomarkers, characterized by their tissue‐specific DNA methylation patterns, have emerged as a promising method for pinpointing the origin of ccfDNA. Despite their potential, further targeted research is essential to deepen our understanding. Unlike tissue biopsies, which risk DNA integrity during preparation, ctDNA samples from plasma/serum remain intact,^194^ allowing for the identification of the tissue of origin through methylation profile differences.^195^, ^196^ Studies like CancerSEEK and Methylation Group GRAIL have underscored ctDNA's capability in identifying the tissue origin in disseminated malignancies without a clear primary focus, achieving up to 83% accuracy.^197^ “Plasma DNA tissue mapping” through genome‐wide analysis supports the feasibility of determining cfDNA tissue origin by analyzing methylation patterns across tissues.^198^ Additionally, research using tissue‐specific DNA methylation biomarkers and digital PCR to measure DNA concentrations in plasma has shown clinical applications in metastatic CRC, particularly for hepatic and colonic origins.^199^ In a study of 26 HCC patients, the association of 5hmC modifications in a select list of genes provided estimates for the origin of these modifications, indicating that 5hmC in cfDNA correlates with tissue origin.^200^ Tian et al.’s^26^ research revealed a strong correlation between differential methylation marks in ENKTL plasma and tissue DNA methylation, suggesting ctDNA methylation patterns could indicate ENKTL tissue methylation status. Given the unique methylation patterns across tissues, ctDNA methylation analysis holds promise for identifying specific tissue sources, offering new avenues for detecting malignancies where traditional biopsy methods are inadequate.

### 4.6 Predictive immunotherapy

Wen et al.^201^ found that the combination of natural killer cell activity and methylation status of HOXA9 ctDNA in NSCLC patients receiving programmed cell death protein 1 (PD‐1)/its ligand PD‐L1 inhibitor therapy has prognostic value. The treatment of diffuse large B‐cell lymphoma (DLBCL) heavily relies on intensive anthracycline‐based chemoimmunotherapy. Tumor cells treated with azacitidine have shown increased sensitivity to chemoimmunotherapy.^202^ Brem et al.^203^ have initiated a randomized trial incorporating oral hypomethylating agents for older DLBCL patients, aiming to reduce injection frequency. This trial also explores methylation patterns in normal T cells and ctDNA to understand the effects of demethylating drugs on both tumor DNA and healthy cells.^203^ In NSCLC, systemic therapy often leads to rapid responses but is marred by drug resistance and vulnerability to distant metastases. The analysis of ctDNA has facilitated the identification of drug resistance linked to CYP2D6 deficiency and the evaluation of treatment responses.^204^ DNA methylation deficiency, associated with chromosomal instability, may predict poor responses to immunotherapy.^205^ Further analysis revealed a positive correlation between CYP2D6 methylation and lymphocyte infiltration in NSCLC samples, suggesting that higher CYP2D6 methylation levels indicate increased immunogenicity. This insight led to the identification of MEK inhibitors as potential treatments for CYP2D6 deficiency.^204^ These findings highlight innovative approaches to overcoming resistance in systemic NSCLC therapy and underscore the importance of advancing new therapeutic strategies. While predicting neoantigens directly remains challenging, ctDNA methylation offers an indirect, probabilistic indicator of immunogenicity, paving the way for personalized cancer treatment.

### 4.7 Clinical trials employing ctDNA methylation detection

ctDNA methylation detection has emerged as a promising tool for early cancer diagnosis, monitoring, and prognosis. This summary focuses on ongoing and completed clinical trials that utilize ctDNA methylation detection across various cancer types, summarized in Table 5 by pathway and mechanism of action. Trials NCT05801263^206^ and NCT05801276^207^ investigate the diagnostic sensitivity and specificity of CDO1 and HOXA9 methylation assays. These studies aim to validate the clinical application of these biomarkers for early detection and prognosis in OC. Additionally, NCT05578625^208^ focuses on monitoring MRD in multiple myeloma (MM) patients using plasma ctDNA methylation sequencing. The primary outcome is the completion of sample collection, with the goal of tracking clonal evolution patterns during disease progression. Furthermore, several trials (NCT05536089,^209^ NCT05558436,^210^ NCT03737591,^211^ NCT06347887,^212^ NCT03828396,^213^ NCT03737539,^214^ NCT05508503,^215^ NCT05904665,^216^ NCT05954078^217^) evaluate the efficacy of ctDNA methylation assays for monitoring postoperative relapse, assessing adjuvant chemotherapy efficacy, and early detection of CRC and adenomas. These studies focus on improving diagnostic accuracy, DFS, and ctDNA clearance rates. Similarly, trials NCT03685669,^218^ NCT03651986,^219^ NCT06358222,^220^ NCT04253509,^221^ NCT03181490,^222^ and NCT03634826^223^ aim to develop noninvasive methods for detecting lung cancer‐specific ctDNA in blood. These studies focus on improving the specificity of early lung cancer screening, differentiating benign and malignant pulmonary nodules, and monitoring postoperative recurrence. Moreover, NCT03483922^224^ aims to develop noninvasive biomarkers based on ctDNA methylation changes for HCC detection. The primary outcome is the calculation of *M* scores and HCC probability scores. Additionally, NCT05858242^225^ investigates the application of ctDNA methylation in early screening, diagnosis, and postoperative monitoring of BC. The study aims to establish a detection system and evaluate the predictive effect of ctDNA methylation on postoperative prognosis. Last, trials NCT03922230^226^ and NCT04511559^227^ focus on detecting hydroxymethylation and ctDNA methylation in esophageal squamous cell carcinoma (ESCC) and gastric cancer, respectively. These studies aim to identify methylation markers associated with early cancer detection and prognosis, with the collective goal of enhancing early cancer detection, improving diagnostic accuracy, and providing better monitoring of cancer recurrence through innovative ctDNA methylation detection methods. The integration of ctDNA methylation analysis with other diagnostic tools holds significant potential for transforming cancer diagnostics and patient management.

**TABLE 5:** Summary of ongoing and completed trials utilizing ctDNA methylation detection.

NCT number	Study type	Enrollment	Sample stage	Action pathway and mechanism	Outcome measures	Sponsor	References
NCT05801263	Observational	5000	OC	Detection of CDO1 and HOXA9 methylation in plasma ctDNA for early OC diagnosis	Diagnostic sensitivity and specificity; PFS	Lei Li	^206^
NCT05801276	Observational	1400	OC	Further exploration of CDO1 and HOXA9 methylation for ovarian cancer diagnosis	Diagnostic sensitivity and specificity; PFS	Lei Li	^207^
NCT05578625	Observational	66	MM	ctDNA methylation sequencing for MRD monitoring in MM	Sample collection completion	Institute of Hematology	^208^
NCT05536089	Observational	2000	Stage I/II CRC	Multilocus ctDNA methylation assay for relapse monitoring and chemotherapy evaluation	5‐year DFS, ctDNA vs. CT/MRI	Singlera Genomics Inc.	^209^
NCT05558436	Observational	662	Colorectal Tumor	Comparison of ctDNA methylation and CEA in detecting CRC	Diagnostic sensitivity; stage stratification performance	Nanfang Hospital	^210^
NCT03737591	Observational	1138	CRC, Adenoma	Evaluation of plasma ctDNA methylation haplotypes in detecting CRC and adenoma	Sensitivity and specificity	Fudan University	^211^
NCT06347887	Observational	1965	CRC	Multigene methylation test (ColonAiQ) for CRC diagnosis	Sensitivity and specificity; PPV; NPV	Singlera Genomics Inc.	^212^
NCT03828396	Observational	1300	CRC, Advanced Adenoma	Detection system based on plasma ctDNA methylation for CRC and adenoma	Assay sensitivity and specificity	Shanghai Zhongshan Hospital	^213^
NCT03737539	Observational	300	CRC	Dynamic monitoring of ctDNA methylation for CRC recurrence prediction	DFS; ctDNA vs. CT/MRI	Fudan University	^214^
NCT05508503	Observational	1378	CRC, Adenoma	Evaluation of a dual‐target (NTMT1 and MAP3K14‐AS1) ctDNA methylation test for CRC and adenoma	Sensitivity, specificity and accuracy	Changhai Hospital	^215^
NCT05904665	Interventional	526	Nonmetastatic CRC	Quantitative ctDNA methylation monitoring for CRC recurrence prediction	Sensitivity, specificity and accuracy	Fudan University	^216^
NCT05954078	Interventional	340	High‐risk stage II, III CRC	Exploration of optimized adjuvant chemotherapy guided by ctDNA methylation	ctDNA clearance rate, DFS, OS	Fudan University	^217^
NCT03685669	Observational	300	Lung Nodule	ctDNA methylation for differentiating benign from malignant pulmonary nodules	Noninvasive diagnosis of benign and malignant nodules	Shanghai Chest Hospital	^218^
NCT03651986	Observational	10560	Pulmonary Nodules	Early differentiation of benign and malignant pulmonary nodules	Diagnostic performance of ctDNA methylation	AnchorDx Medical Co., Ltd.	^219^
NCT06358222	Observational	200	NSCLC	Prediction of NSCLC lymph node metastasis based on ctDNA methylation	NPV; DFS; CFI; OS	Shanghai Chest Hospital	^220^
NCT04253509	Observational	280	LC	Investigation of ctDNA methylation sequencing in LC diagnosis	Diagnostic sensitivity, specificity and accuracy; PPV; NPV	Samsung Medical Center	^221^
NCT03181490	Observational	1490	Pulmonary Nodule	Validation of ctDNA methylation test for early LC diagnosis	Diagnostic performance	The First Affiliated Hospital of Guangzhou Medical University	^222^
NCT03634826	Observational	200	LC	Longitudinal monitoring of ctDNA methylation in LC patients	Correlation between recurrence and ctDNA	Peking University People's Hospital	^223^
NCT03483922	Observational	403	HCC	ctDNA methylation in PBMC and ctDNA for HCC diagnosis and prognosis	Calculation of *M* scores and HCC probability scores	HKGepitherapeutics	^224^
NCT05858242	Observational	100	BC	Early screening and diagnosis of BC using ctDNA methylation	Characteristic ctDNA methylation targets	Singlera Genomics Inc.	^225^
NCT03922230	Observational	300	ESCC	Genomic hydroxymethylation analysis for early ESCC screening	Hydroxymethylation level	Nanfang Hospital	^226^
NCT04511559	Observational	540	GC	Correlation of ctDNA methylation with diagnosis and prognosis of GC	ctDNA methylation status	Shanghai Zhongshan Hospital	^227^
NCT03868215	Observational	100	CRC, Lymph Node Metastases	Detection of local residual or lymph node metastasis using ctDNA methylation haplotypes	Sensitivity of ctDNA methylation	Fudan University	^228^
NCT06167967	Interventional	990	CRC	Randomized controlled trial investigating ctDNA methylation‐guided adjuvant chemotherapy in CRC	RFS; OS; ctDNA clearance rate	Sir Run Run Shaw Hospital	^229^

Abbreviations: CFI, ctDNA‐free interval; CT, computed tomography; ESCC, esophageal squamous cell carcinoma; MM, multiple myeloma; MRI, magnetic resonance imaging; NPV, negative predictive value; PPV, positive predictive value.

These studies highlight the pivotal role of ctDNA methylation and liquid biopsy in early cancer detection, prognosis evaluation, and disease monitoring. The advancement of these technologies offers novel tools and approaches for precise diagnosis and personalized medicine in cancer care.

Although ctDNA methylation detection holds promise, several challenges must be addressed before its widespread adoption in clinical practice. Key challenges include developing reproducible assays with high sensitivity and specificity and validating biomarker efficacy in prospectively collected target populations. The determination of the optimal panel for various stages of cancer diagnosis, recurrence monitoring, treatment response prediction, and prognosis, given the proven utility of specific promoter regions, remains unresolved. Identifying methylation markers that combine satisfactory sensitivity and specificity with the capability for rapid detection in large sample sizes is essential. The development of prognostic methylation markers faces numerous obstacles, partly due to difficulties in standardizing detection techniques across studies and identifying universally accepted prognostic methylation sites. Moreover, current detection methods often rely on sulfite conversion, which may damage ctDNA.^230^, ^231^ It is crucial to acknowledge that DNA methylation patterns vary across different ethnicities and environmental exposures before applying ctDNA methylation assays in clinical settings. The presence of ctDNA methylation in a significant number of healthy controls,^232^, ^233^ along with the potential for false positives or preoperative false negatives,^82^ necessitates further optimization. Effective screening methods must achieve high specificity to minimize the risk of overdiagnosis,^234^ especially given the low incidence of cancer. The manual nature of current assays, including sample preparation, DNA extraction, and bisulfite conversion, introduces the risk of errors and inefficiencies. The reliance on retrospective data and the lack of serial plasma samples in several studies^25^, ^66^, ^147^, ^235^ call for validation in larger, more homogeneous cohorts with consistent follow‐up to ensure the robustness, reproducibility, and reliability of reported results. The high cost of sequencing per sample poses another barrier to cost‐effective screening, highlighting the need for simpler, more affordable methods for identifying and measuring methylation levels. Observational studies often suffer from variability in treatment and follow‐up protocols, limiting their applicability compared with structured prospective clinical trials. Ultimately, the full clinical application of ctDNA methylation detection depends on demonstrating its clinical validity and utility, paving the way for its significant impact on genomic oncology and the management of cancer patients.

## 5 CONCLUSION AND PERSPECTIVES

The detection of ctDNA methylation has emerged as a promising biomarker for liquid biopsy in cancer diagnosis, prognosis, and disease monitoring. The unique advantages of ctDNA methylation, including its early appearance, cancer specificity, and biological stability, make it an attractive alternative to traditional tissue biopsies. The comprehensive coverage of genome‐wide methylation patterns by techniques such as WGBS has enabled sensitive and specific detection of ctDNA methylation markers across various cancer types. The integration of machine learning algorithms in analyzing ctDNA methylation data has addressed the challenge of low ctDNA levels in plasma samples, further enhancing the detection accuracy. The clinical application of ctDNA methylation has shown promising results in early cancer detection, tumor recurrence monitoring, and personalized treatment response prediction. In particular, the ability of ctDNA methylation analysis to track tumor burden and monitor treatment efficacy in real‐time offers significant advantages over imaging techniques.

This review explores the application of ctDNA methylation in tumor liquid biopsy, highlighting methylated ctDNA markers as a promising approach for cfDNA screening and a potential alternative to tissue biopsy. The advantages of early detection, cancer specificity, biological stability, and accessibility in body fluids^230^ make ctDNA methylation particularly appealing. The incorporation of machine learning to analyze abnormal DNA methylation addresses the challenge of limited ctDNA levels in plasma samples. Furthermore, standardizing DNA extraction and PCR techniques is essential for ensuring consistent results across studies. Future research should expand to include diverse sample types such as CSF, saliva, pleural fluid, and urine, alongside plasma and serum. Although ctDNA methylation may not immediately replace current diagnostic standards, it represents a promising direction for cancer detection and monitoring. Tao et al.^236^ analyzed multiomics data from patients with CRC and gastric adenocarcinoma, finding it superior to mono‐omics data in detecting tumor genes and identifying suppressed peripheral immune features in cancer patients. Integrating ctDNA methylation detection with exosomes,^237^, ^238^ circulating miRNA, CTCs, metabolomics, molecular imaging, and other multiomics analyses could significantly enhance diagnostic effectiveness and clinical utility. Comprehensive studies across multiple centers are essential to fully understand the clinical applications of these methods in improving cancer treatment and prognosis. Looking ahead, assays capable of detecting a broad spectrum of mutations and gene hypermethylation may become the most effective means of leveraging ctDNA for global cancer diagnosis and monitoring. The convergence of deep learning and data analysis promises to provide a powerful tool for the early identification of cancer and the classification of its subtypes on a genome‐wide scale. With the decreasing cost of NGS, ctDNA–WGBS is poised for broader application in both basic and clinical research, paving the way for advancements in the field of oncology.

In recent years, ctDNA methylation, particularly in CRC, has undergone extensive research, demonstrating high sensitivity and specificity in SEPT9 methylation analysis—100 and 97%, respectively.^124^ Enhancing ctDNA testing specificity could significantly reduce future healthcare costs, protect patients from unnecessary invasive procedures, and lessen patient anxiety. Beyond blood, the feasibility of employing ctDNA methylation analysis for detecting nonmetastatic NSCLC in urine has been explored,^133^ suggesting potential for broader diagnostic applications. The association of DNA methylation with other ctDNA abnormalities, such as mutations, copy number variations, or differences in fragment length,^239^ underscores its potential to improve cancer detection efficacy in urine samples.^240^, ^241^ Accurate biomarker specificity determination necessitates a large control group without cancer, ideally matching in age distribution and collected under standardized conditions. This review underscores the potential of methylated ctDNA as a circulating biomarker for cancer diagnosis, monitoring recurrence, and predicting treatment outcomes. With thorough clinical trials and validation, these biomarkers could significantly advance early cancer detection and patient management. Optimizing and standardizing the extraction and quantification of ctDNA from blood samples, alongside performance evaluation as biomarkers, are crucial steps toward their clinical adoption. Furthermore, refining algorithms or scoring systems to account for confounding factors could bolster the reliability and effectiveness of these biomarkers in clinical applications.

Looking ahead, the clinical application of ctDNA methylation detection is poised for further expansion. With the decreasing cost of NGS and the continued development of more sensitive and specific detection methods, ctDNA–WGBS is expected to become more accessible and widely used in both basic and clinical research. Integration of ctDNA methylation detection with other liquid biopsy technologies, such as CTC analysis and EV analysis, could further enhance diagnostic precision and clinical utility. Moreover, the identification of novel methylation markers and the refinement of existing marker panels will be crucial for advancing ctDNA methylation‐based diagnostics. With the growing availability of multiomics data, comprehensive studies across multiple centers will be essential to fully understand the clinical implications of ctDNA methylation patterns and their role in cancer management. In conclusion, ctDNA methylation detection represents a significant step forward in the field of liquid biopsy and has the potential to revolutionize cancer diagnostics and treatment. The integration of this technology into routine clinical practice could significantly improve patient outcomes and reduce healthcare costs by enabling early detection, personalized treatment, and effective disease monitoring.

## 6 HIGHLIGHTS

ctDNA methylation detection stands out as both a sensitive and cost‐effective liquid biopsy technique, offering significant advantages for the clinical management of cancer. ctDNA uniquely mirrors the genetic composition of its originating tumor tissue, providing a broader spectrum of information than traditional biopsies due to tumor heterogeneity. Its rapid degradation rate makes ctDNA an ideal marker for ongoing monitoring, allowing for the real‐time assessment of tumor burden and the effectiveness of treatments. Beyond these benefits, ctDNA methylation analysis surpasses somatic mutation detection in sensitivity, detection range, and the ability to target multiple regions.^242^ This method enables the classification of tumors based on distinct methylation patterns across different tissues. To date, several methylation markers, such as SEPT9 for CRC,^243^ have been clinically validated. Research by Cao et al.^27^ highlighted the limitations of AFP alone in detecting HCC, suggesting ctDNA methylation as a valuable adjunct to ultrasound plus AFP for HCC detection. Integrating various biomarker strategies could potentially offer novel perspectives in the diagnosis of HCC. Furthermore, tumor‐agnostic approaches, unaffected by tumor accessibility or cellularity, promise to refine patient management. Employing TriMeth or similar ctDNA assays could improve the stratification of CRLM surveillance, offering a nuanced risk assessment beyond traditional markers like CEA.^77^ This indicates the potential of ctDNA methylation detection not just in improving diagnostic precision but also in tailoring patient‐specific treatment strategies, marking a significant advancement in personalized oncology care.

ctDNA detection offers a noninvasive, repeatable method that can be utilized at any stage of treatment, boasting greater sensitivity than imaging for identifying minimal residual tumors.^244^, ^245^, ^246^ Consequently, ctDNA emerges as a crucial marker for dynamically monitoring disease progression.^246^ Specifically, the mSEPT9 DNA marker, with a sensitivity of 72.94% and specificity of 81.97%, outperforms traditional markers like CEA and CA19‐9 in differentiating CRC patients from healthy controls.^247^ Further research has shown enhanced sensitivity in monitoring CRC recurrence when mSEPT9 detection is integrated with imaging techniques.^156^ Early detection of changes in BCAT1 and IKZF1 in plasma, even before radiological signs of recurrence,^152^, ^168^ highlights ctDNA's potential in identifying clinically significant recurrences, especially following potentially curative surgeries. The study by Song et al.^171^ underscored mSEPT9's superiority over CEA in three key aspects of evaluating surgical outcomes: a higher proportion of patients could be assessed using mSEPT9, reflecting its higher preoperative detection sensitivity; postoperative mSEPT9 levels showed a more significant decrease than CEA, aiding in clearer differentiation of treatment effects; and a positive correlation was observed between tumor size and mSEPT9 levels, suggesting its utility as a quantitative marker for monitoring tumor progression or regression, a correlation not seen with CEA. Unlike serum protein markers for CRC, which lack a significant quantitative relationship with disease progression, methylation markers generally exhibit a quantitative correlation with the disease's advancement.^232^, ^248^ This is attributed to the amount of ctDNA released into the bloodstream by tumors, which typically reflects tumor size and lesion severity, a relationship less apparent in protein markers. However, the utility of plasma ctDNA is limited for brain tumors due to the blood‐brain barrier.^249^ CSF, closely associated with malignant brain tumors and known to contain ctDNA,^250^ presents a more viable source for ctDNA in these cases. Thus, CSF may offer a superior alternative for real‐time monitoring of disease progression and treatment efficacy in brain tumor patients. While most ctDNA analyses in CSF have focused on detecting cancer‐associated mutations, the prevalence of epigenetic alterations in pediatric brain tumors suggests that analyzing epigenetic markers in CSF ctDNA could provide a precise and sensitive means of evaluating treatment response, disease progression, and recurrence in brain tumors.

One of the critical advantages of ctDNA methylation detection is its potential to overcome the limitations of traditional tissue biopsies. Tissue biopsies, although informative, are invasive and may not fully represent the genetic heterogeneity of tumors. In contrast, ctDNA methylation patterns mirror those in tumor tissues, providing a comprehensive picture of tumor genetics without the need for invasive procedures. This noninvasive nature of liquid biopsy makes it particularly attractive for early cancer screening and longitudinal monitoring of tumor dynamics. Another significant finding is the role of ctDNA methylation in predicting treatment response and prognosis. Changes in ctDNA levels have been shown to correlate with treatment outcomes, enabling real‐time monitoring of disease progression and facilitating timely intervention. This capability has significant implications for personalized cancer management, as it allows for the tailored adjustment of treatment strategies based on individual patient responses.

## AUTHOR CONTRIBUTIONS

Yingli Sun was responsible for the design and revision of this paper. Lingyu Li wrote in full, including text, tables and figures, and modified. All the authors reviewed and approved the final manuscript.

## CONFLICT OF INTEREST STATEMENT

The authors declare no conflict of interests.

## ETHICS STATEMENT

Not applicable.

## Data Availability

Not applicable.
